# Complex patterns of cell growth in the placenta in normal pregnancy and as adaptations to maternal diet restriction

**DOI:** 10.1371/journal.pone.0226735

**Published:** 2020-01-09

**Authors:** Malcolm Eaton, Alastair H. Davies, Jay Devine, Xiang Zhao, David G. Simmons, Elín Maríusdóttir, David R. C. Natale, John R. Matyas, Elizabeth A. Bering, Matthew L. Workentine, Benedikt Hallgrimsson, James C. Cross

**Affiliations:** 1 Department of Biochemistry and Molecular Biology, Cumming School of Medicine, University of Calgary, Calgary Alberta; 2 Department of Comparative Biology and Experimental Medicine, Faculty of Veterinary Medicine, University of Calgary, Calgary Alberta; 3 Department of Anatomy and Cell Biology, Cumming School of Medicine, University of Calgary, Calgary Alberta; 4 Faculty of Veterinary Medicine, University of Calgary, Calgary Alberta; Brigham and Women's Hospital, UNITED STATES

## Abstract

The major milestones in mouse placental development are well described, but our understanding is limited to how the placenta can adapt to damage or changes in the environment. By using stereology and expression of cell cycle markers, we found that the placenta grows under normal conditions not just by hyperplasia of trophoblast cells but also through extensive polyploidy and cell hypertrophy. In response to feeding a low protein diet to mothers prior to and during pregnancy, to mimic chronic malnutrition, we found that this normal program was altered and that it was influenced by the sex of the conceptus. Male fetuses showed intrauterine growth restriction (IUGR) by embryonic day (E) 18.5, just before term, whereas female fetuses showed IUGR as early as E16.5. This difference was correlated with differences in the size of the labyrinth layer of the placenta, the site of nutrient and gas exchange. Functional changes were implied based on up-regulation of nutrient transporter genes. The junctional zone was also affected, with a reduction in both glycogen trophoblast and spongiotrophoblast cells. These changes were associated with increased expression of *Phlda2* and reduced expression of *Egfr*. Polyploidy, which results from endoreduplication, is a normal feature of trophoblast giant cells (TGC) but also spongiotrophoblast cells. Ploidy was increased in sinusoidal-TGCs and spongiotrophoblast cells, but not parietal-TGCs, in low protein placentas. These results indicate that the placenta undergoes a range of changes in development and function in response to poor maternal diet, many of which we interpret are aimed at mitigating the impacts on fetal and maternal health.

## Introduction

The placenta is a complex organ that integrates the fetal demand for nutrients with maternal nutrient availability [1], while also protecting the health of the mother [2]. Deficits in the maternal environment limit nutrient and oxygen availability, often resulting in intrauterine growth restriction (IUGR) [3–5] resulting in low birth weight, increased risk of perinatal complications, and increased long-term risk of cardiovascular and metabolic diseases such as type II diabetes [6,7]. Despite this, not all pregnancies complicated by poor maternal environment result in IUGR. Several studies in experimental animals show that the placenta is highly adaptable, changing its development and/or physiology to delay or mitigate the effects of suboptimal resource availability [2]. In humans, time-course analysis is restricted to gross measures of fetal and placental physiology, making animal models a critical tool for answering questions about the effects of maternal environment on fetal and placental development [8,9].

Although the general phases of normal placental growth and development are well known [10–13], the underlying cellular dynamics are less well understood. The mechanisms underlying the ability of the placenta to adapt its structure and function via cellular adaptations in the face of a suboptimal maternal environment is even less clear. The mouse placenta is comprised of three distinct zones; the decidua which is maternal in origin, and the conceptus-derived junctional and labyrinth zones. The junctional zone is made up of parietal trophoblast giant cells (P-TGCs), as well as glycogen trophoblast (GlyT) and spongiotrophoblast (SpT) cells [12]. GlyT cells store energy in the form of glycogen that is thought to buffer energy demands during late pregnancy [10,14]. SpT cells are believed to be primarily endocrine in nature [15,16] and may drive maternal metabolic adaptations to pregnancy that alter nutrient availability to the placenta. The labyrinth zone is composed of two layers of multinucleated syncytiotrophoblast (SynT) cells that form the site of nutrient and gas exchange and underlying fetal vasculature. In the mature placenta, the maternal blood spaces in the labyrinth are not lined by endothelial cells, but rather by conceptus-derived sinusoidal trophoblast giant cells (S-TGC) that are also endocrine cells. Normal maturation and potentially adaptive changes in the labyrinth zone in response to altered diet are driven in part by the *Igf2* gene, which regulates interhemal thickness and nutrient transporter expression [17]

While many studies have described the overall size of the distinct placental zones, none have considered the relative contributions of cell proliferation versus cell hypertrophy to the growth of the placenta during gestation. Cell size is correlated with DNA content, and cells become polyploid through endoreduplication, the process by which cells undergo rounds of DNA replication without intervening mitoses. Endoreduplication occurs in several trophoblast subtypes in the mouse placenta during normal development [12], particularly TGCs. P-TGCs can contain up to 1000 copies of DNA in the same cell [18], though interestingly, P-TGCs do not uniformly replicate their genomes. Certain regions of the P-TGC genome are consistently under-replicated [19], whereas other loci, including those encoding placenta-specific polypeptide hormones, are consistently over-replicated [20].

All the above-mentioned processes are subject to change, given differences in the fetal and maternal environment. The effects of an isocaloric low protein diet on placental and fetal growth are highly variable, and depend on the model (e.g., rat, mouse, sheep) and the timing of protein restriction [21–26]. Experimental studies have shown that feeding pregnant females an isocaloric low protein diet increases the incidence of cardiovascular disease in the offspring postnatally, particularly in males [27]. Using a mouse model of chronic protein restriction beginning two weeks prior to mating and maintained during gestation, to mimic chronic malnutrition in humans, we found previously that fetal weight in the protein-restricted group was no different than controls until after embryonic day (E) 17.5, just before term. By contrast, placenta growth was diminished as early as E10.5 in the low protein group and mothers lost weight before E17.5. The different zones of the placenta were not affected to the same extent, with the junctional zone contributing most to the size variation [28]. There was a reduction in the GlyT cell population in the protein-restricted group but whether this reflects a pathological or adaptive response is unknown. In the previous study, there was no direct measure of SpT cells but expression of *Prl3a1*, a placental prolactin-related hormone gene that is expressed exclusively in SpT cells was affected. Interestingly, expression of *Prl3a1* mRNA was upregulated before E17.5 when fetuses were still growing normally but sharply decreased by E18.5 when fetuses became growth restricted.

How genetic and environmental perturbations give rise to cellular variation in the placenta is a critical question but to date it has often been investigated without taking the sex of conceptus into account. Sex is an important factor that, when ignored, could mask certain interactions affecting placental adaptations [29,30]. For example, male placentas are significantly heavier than female placentas [31] and it is known that differences in placenta size co-vary with morphological and functional adaptations [32]. Hence, it is no surprise that sexually divergent gene expression and methylation patterns in the placenta have been demonstrated in response to feeding mothers diets that are high- or low-fat [33,34] and in cases of caloric restriction [35]. Differences in placental gene expression are also associated with birthweight disparities between sexes in calorie and protein restriction models [36,37]. Postnatal effects of altered maternal diet during pregnancy are also sexually dimorphic, with differences in catch-up growth and metabolism that affect post-natal life [36].

The objectives of our current study were first to describe the patterns of cell proliferation and endoreduplication during the time course of normal gestation and then to see how these normal patterns of development and function are affected by a chronic low protein maternal diet.

## Materials and methods

### Animals

The work was approved by the University of Calgary Health Sciences Animal Care Committee (AC12-0094). Mice were housed under normal laboratory conditions with a 12-hour light: 12-hour dark photoperiod and unrestricted access to food and water. The presence of a vaginal plug was defined as embryonic day (E) 0.5. For studies of normal development, CD1 mice (Charles River) were mated and pregnant females were dissected every two days, beginning at embryonic day (E) 8.5 through E18.5. C57BL/6 mice were used to assess the impact of a low protein diet during pregnancy. Four-week-old female mice (Charles River) were maintained on a 12-hour light: 12-hour dark cycle for four weeks. Nulliparous females were randomly divided into two groups and fed a control (Con) (20%) (Envigo cat. No. TD.91352) or low protein (LP) (6%) (Envigo cat. No. TD.90016) diet for two weeks before mating. After mating, females were maintained on their respective diets until sacrifice. The diets were both iso-caloric (3.8 Kcal/g) but differed in the percentage of protein in the form of casein and DL-Methionine. Fetuses and placentas were dissected and weighed on E13.5, 16.5, and 18.5 (day before delivery). All animal care and procedures were conducted in compliance with the University of Calgary Health Sciences Animal Care Committee and with the guidelines of the Canada Council on Animal Care (Protocol AC16-0089).

Maternal weight was recorded before and after dissection of the uterus. Fetal and placental weights were measured immediately after dissection of the uterus and surrounding tissue. Placental efficiency was estimated by dividing individual fetal weights by the corresponding placental weight.

### Sex determination

A small section of the tail was removed from each fetus and DNA was extracted using tissue preparation, extraction, and neutralization solutions (Sigma). Each sample was PCR amplified and the presence of a region of the *Sry* gene (249 bp) indicated a Y chromosome. Amplification of a ~400 bp sequence in the *Hand1* gene was used as a positive control to allow detection of PCR inhibition and failure of extraction.

### Tissue processing

Following dissection and weighing, placentas were hemisected and immediately fixed in 4% paraformaldehyde (PFA) at 4°C overnight. Tissues were then washed in 1 x phosphate-buffered saline (PBS), processed through increasing concentrations of sucrose solutions (10%, 20%), and finally embedded in Tissue-Tek O.C.T. compound (Electron Microscopy Sciences). Placentas were sectioned using a Leica CM3050S cryostat, and 20 μm sections were cut, transferred to Superfrost Plus slides (Fisher Scientific), and stored at -80°C. To remove bias, systematic random sampling was used to select sections for further analysis [38].

### Immunofluorescence staining

Double immunofluorescence staining was used to identify proliferating cells throughout placental sections: specifically, trophoblast, phospho-histone H3/Cytokeratin 18 (K18) double-positive, endothelial, phospho-histone H3/PECAM1 double-positive and pericyte, phospho-histone H3/alpha-smooth muscle actin (Acta2) double-positive cells. Three sections from three placentas at each gestational time point were analyzed. Sections were thawed to room temperature and rinsed in 1X PBS. Subsequently, they were heated in a 0.01 M sodium citrate buffer (pH 6.5) to expose antigens before blocking in 5% goat serum (Cedarlane) in 1X PBS/1% BSA for 45 minutes. Primary antibodies (anti-phospho-histone H3, 1:100; anti-K18, 1:100; Fitzgerald; anti-phospho-histone H3, 1:100; Upstate; anti-PECAM, 1:300; Abcam and anti-Acta2, 1:300; Sigma) were applied stepwise in the appropriate combinations to the cryosections and incubated overnight at 4°C. After washing, sections were incubated for one hour with secondary antibodies (Alexa488-conjugated goat anti-mouse, 1:300; Alexa488-conjugated anti-rat, 1:300; both Molecular Probes; 1:300 or cy3-conjugated goat anti-rabbit, 1:300; Jackson Immuno Research), depending on primary antibody combinations. Nuclear staining was carried out using 25 mg/ml bisbenzimide (Sigma). Ki67/K18 double-immunofluorescence staining was carried out using the same procedure as described above using rabbit polyclonal anti-Ki67 (Abcam; 1:50) and human monoclonal anti-K18 (Fitzgerald; 1:100) primary antibodies.

### Calculation of cell cycle length

The length of the cell cycle in different trophoblast cell subtypes was estimated two ways: 1) from the change in cell number over the time course of study; 2) the number of phospho-histone H3/K18 and Ki67/K18 double-positive cells as follows:

$$Length\;of\;total\;cell\;cycle=\;\frac{Ki67\;positive\;cells\;\left(\%\right)\times Length\;of\;G2/M\;phase}{Phosphohistone\;H3\;positive\;cells\;\left(\%\right)}$$

The length of the G2/M phase was assumed to be 0.8 hours, true for most mammalian cells.

### In situ hybridization

Fixed placentas were dehydrated through ethanol and xylene gradients, then embedded in paraffin. *In-situ* hybridization was performed according to [39]. For *in situ* hybridization at least 3 sections from at least three male and three female placentas were analyzed for each diet group and developmental stage. Placentas were bisected near the midline, and at least 80 seven μm sections were obtained. At least three sections within 240 μm of the estimated midline were sampled and analyzed for each diet, sex, and gestational age group combination. The borders of the three main placental layers were distinguished by performing in situ hybridization for *Tpbpa* mRNA, which is expressed in SpT and GlyT cells. SpT and GlyT cells were distinguished by expression of *Prl8a8* mRNA and *Pcdh12* mRNA, respectively. After *in situ* hybridization, these slides were counterstained with DAPI. Brightfield and fluorescent images were taken with a widefield microscope (Thorlabs Tide whole-slide scanner). Total SpT and GlyT cell area were estimated by manually tracing the area expressing *Prl8a8 and Pcdh12* mRNA, respectively, using ImageJ. To ensure cell specificity, the *Prl8a8* expressing area was superimposed onto the corresponding DAPI image. Cell number was then determined using a thresholding and particle analysis macro written for ImageJ. Cell size was estimated by dividing the total cell area by the total number of cells quantified.

### Estimation of DNA content

Tissue sections were deparaffinized and placed in DAPI (1mg/ml) diluted 1:5000 in 1X PBS for four minutes, followed rinsing in 1X PBS for two minutes with slight agitation. Coverslips were mounted immediately with Fluoromount-G mounting solution (Southern Biotech, Birmingham, AL) on Superfrost+ slides (VWR) and sections were stored at 4°C overnight in the dark. Images were acquired on the Zeiss Axioimager at 20x magnification. Slides were imaged in random order, and imaging parameters were held constant throughout, ensuring that fluorescent intensity covered the full dynamic range of the camera. At least three randomly-chosen regions containing P-TGCs and SpT cells were imaged, along with two images of the labyrinth zone. P-TGCs were easily distinguished by their large nucleus. SpT cells were identified as cells between P-TGCs and the labyrinth. To distinguish between SpT cells and Gly-T cells, nuclei between 80–200 μm^2^ were sampled. S-TGCs are the only TGC subtype in the labyrinth and were therefore easily distinguished by nuclear size. Ten P-TGCs, 10 S-TGCs, and 20 SpT cells were randomly selected and their nuclei manually traced in ImageJ. The ploidy of each trophoblast cell type was estimated by dividing the corrected total nuclear fluorescence (CTNF) of each cell to the CTNF of amniotic epithelial cells (diploid = 2C) on the same slide. CTNF was calculated as follows:

CTNF=Integrateddensity−(nuclearareaxmeanbackgroundfluoresence)

### Stereology

Three sagittal, non-adjacent sections evenly spaced by 70 μm from the estimated midline of the placenta were stained for alkaline phosphatase activity and counterstained with nuclear fast red (Vector Labs, Burlingame, CA). Alkaline phosphatase activity is expressed on the apical border of SynT (layer I), lining maternal blood sinuses after E12.5 [40]. For each conceptus, four different images of the labyrinth were taken at 40x magnification. A 10x10 point grid was superimposed onto each image (S1 Fig). The presence of either maternal blood space, fetal blood space, S-TGC, SynT cell, fetal endothelial/pericyte, or unknown cell type was scored independently at each point. The percentage of points that overlapped each element was compared between groups. Images were quantified by two blinded investigators and only group differences that remained significant between both observers were considered.

### Real-time qPCR

Three male and three female placentas from at least three litters, in both wildtype and low protein pregnancies, were dissected to isolate RNA (n = 36). Placentas were immersed in 700 ml of Trizol, sonicated, and stored at -80°C until RNA extraction. RNA was extracted using RNeasy Mini kits (Qiagen) according to the manufacturer’s protocol. 1 μg/μL total RNA was extracted from each placenta and RNA from 3 placentas/litter were pooled. Pooled RNA was then reverse-transcribed to cDNA, and each sample diluted to 1:5 in RNase-free water for gene expression analysis using real-time qPCR. Primer sequences are shown in S1 Table. Relative gene expression was measured using the delta-delta Ct method, with samples normalised to the housekeeping gene *Hprt1*, which is stably expressed between samples. Raw Ct values were normalized to *Hprt1* mRNA and converted to Log(1+x) values. Control samples were then normalized to one, and relative expression in LP samples expressed in relation to this. Quantitative analysis was performed on the Quant Studio 6 (Applied Biosystems) using detection with SYBR green. Each sample was analysed in triplicate and probes were verified to have efficiencies between 0.90 and 1.10, followed by sequencing and BLAST analysis to verify target specificity.

### RNA sequencing

RNA from whole placenta was extracted by using Trizol and total RNA was derived from three same-sex littermates (n = 5). TruSeq Stranded mRNA (Illumina) was used with 1 μg of total RNA for the construction of sequencing libraries. cDNA libraries were prepared for sequencing using standard Illumina protocols. Data was deposited in Gene Expression Omnibus (GEO accession GSE131729; https://www.ncbi.nlm.nih.gov/geo/query/acc.cgi?acc=GSE131729). Transcripts were quantified with Kallisto 0.43.1 [41] using *Mus musculus* GRCh38 (Ensembl release 90) cDNA, with sequence bias correction turned on and 50 bootstraps. All downstream analysis was done in R 3.5.1 (R core team 2014). Genes affected by diet (while controlling for gender) were identified using Sleuth 0.30.0 [41]. A Wald test was used to identify transcripts differentially abundant, and the p-values from the transcripts were aggregated for each gene using the Lancaster method as described in [42], in order to generate a gene-level analysis. Differentially expressed genes were selected based on a corrected p-value cutoff of <0.05. Enriched gene sets for both Gene Ontology terms (GO) and Reactome 64 [43] pathways were identified with over-representation tests (p < 0.05) using cluster Profiler 3.10.0 [44] and ReactomePA 1.26.0 [43] respectively. In addition, network plots were generated in GeneMania [45], selecting for genes that were differentially expressed (q <0.01) and associated with the MGI Phenotype terms: “abnormal placenta junctional zone morphology” or, “abnormal labyrinth morphology”.

### Statistical analysis

Cell count data from each zone of the placenta was analyzed using a one-way analysis of variance (ANOVA) followed by Tukey’s honestly significant difference test to determine differences between gestational groups and uncover homogeneous populations. Fetal, placental, and maternal biometry were analyzed using a series of analysis of covariance (ANCOVA) tests. After initializing each ANCOVA as a full model containing every predictor, the model underwent stepwise backward selection until an optimal model with non-significant interaction terms removed. Under the null hypothesis that the group means were equal, a p-value < 0.05 was considered significant. While the dependent variable was either fetal weight, placental weight, or maternal weight, the explanatory variables included diet, sex, placental weight (exclusively for the fetus), and their interactions. To control for interpregnancy variation, litter size and maternal weight were specified as covariates. In addition, the predictors were centred on a mean of zero, because differences in predictor scaling can produce misleading coefficients and interpretations. For other analyses where the normality and homogeneity of variance assumptions were violated within stages and between groups, bootstrapping with replacement was performed. The bootstrapped statistics were regression coefficients, using diet and sex as predictors. A series of resampled (N = 10000) mean coefficient distributions were generated for each effect under the null hypothesis that the difference between groups was equal to zero. A 95% confidence interval and corresponding p-value were reported for each bootstrapped statistic. All multivariate and resampling procedures were performed in R (R Core Team, 2014).

## Results

### Patterns of growth in the mouse placenta during gestation

**Fig 1:**
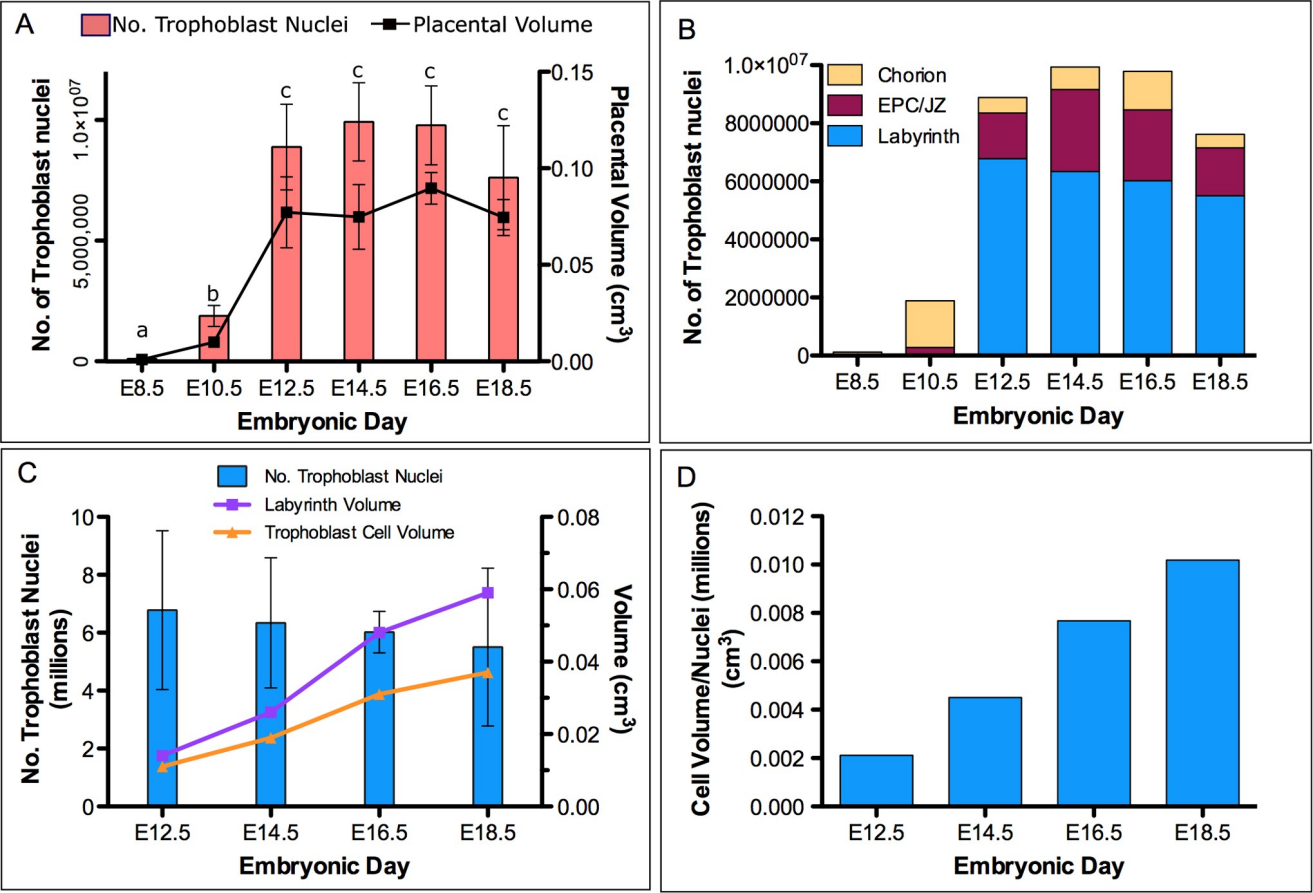
Number of trophoblast nuclei in each zone of the mouse placenta as determined using stereology. (A) Absolute number of trophoblast cell nuclei and total placenta volume. (B) Distribution of trophoblast cell nuclei among different layers of the placenta. (C) Number of trophoblast nuclei within the labyrinth layer (blue bars), as well as labyrinth volume (purple line) and trophoblast cell volume (orange line) from E12.5 to E18.5. (D) Trophoblast cell volume calculated as total trophoblast cell volume in the labyrinth (from C) divided by the number of trophoblast nuclei from E12.5 to E18.5. Different letter labels indicate significant differences between days (p<0.05) and are valid for both volume and total trophoblast number (n = 3).

Stereology was employed to estimate the volume of the normal placenta and its composition throughout gestation because it allows for simple, precise, and unbiased extraction of three-dimensional structural composition and spatial arrangement from two-dimensional histological sections [46]. Absolute placenta volume reached a plateau at E16.5 (Fig 1A), though the rate of placenta volume expansion was not uniform. A dramatic increase in volume of approximately 55-fold occurred between E8.5 and E12.5 compared to a mere 1.4-fold increase in volume between E12.5 and E16.5. The dramatic volume expansion of the placenta between E8.5 and E12.5 was complemented by an increase in total trophoblast cell number from 1.3 X 10^5^ to 9.2 X 10^6^. However, the number of trophoblast cells did not change between E12.5 and E16.5 and then cell number decreased between E16.5 and E18.5 (Fig 1A). Between E10.5 and 12.5, the number of cells in the ectoplacental cone/junctional zone increased by ~10-fold (Fig 1B). The number of cells in the chorionic plate decreased by ~4-fold, but this was balanced by the appearance of trophoblast cells in the labyrinth layer as the chorion trophoblast cells differentiate.

We quantified the proportion of trophoblast cells undergoing the cell cycle using antibodies against Ki67 and phospho-histone H3. Ki67 is a DNA polymerase associated protein that is expressed during early G1 and S phase of the cell cycle [47]. To distinguish between mitotically active cell populations and cell populations that were undergoing endoreduplication, we used double immunofluorescence staining for phospho-histone H3 and keratin 18 (K18). Histone H3 phosphorylation at serine 10 is closely linked to chromosome condensation, a phenomenon that occurs during late G2 through to M phase of the cell cycle [48]. K18 is an intermediate filament protein that is expressed exclusively in cells of trophoblast lineage at the implantation site [49]. Prior to E12.5, 86% and 73% of K18-positive trophoblast cells were Ki67-positive in the chorion and ectoplacental cone, respectively (Table 1). In the chorion, 5.9% of K18-positive cells were phospho-histone H3-positive compared to 3.3% in the ectoplacental cone (Table 1). The large difference between the proportion of Ki67 and phospho-histone H3 staining reflects that fact that the Ki67 epitope is expressed throughout G1/S-phase the cell cycle (~10 hours in chorion trophoblast cells) compared to the phospho-histone H3 epitope which is only expressed during late G2 and M-phase (~0.8 hours). Mitotic cell cycle progression in the chorion and ectoplacental cone was supported by the striking increase in trophoblast cell number. Specifically, in a mere 48 hours, the number of cells in the chorion increased from ~7.8 X 10^4^ to ~1.3 X 10^6^ whereas the number of cells in the ectoplacental cone increased from ~5.3 X 10^4^ to ~2.9 X 10^5^. The rate of mitotic cell cycle progression was not uniform between the chorion and ectoplacental cone. Cell number in the chorion increased at a rate greater than that of the ectoplacental cone, and thus, both the absolute number and proportion of chorion cells exceeded those of the ectoplacental cone (Fig 1B). This trend was substantiated by estimations of cell cycle length (S2 Table). At E8.5, cells in the chorion and ectoplacental cone were, on average, undergoing a complete cell cycle in 9.9 hours and 14.8 hours, respectively.

**Table 1:** Percentage of trophoblast cells undergoing the cell cycle between E8.5 and 18.5. Data represent mean and SEM is in parentheses.

Ki67 as percent of K18-positive cells	E8.5	E10.5	E12.5	E14.5	E16.5	E18.5	ANOVA (*P*)
**Chorion/ Chorioallantoic plate**	87.3%^a^	84.5%^a^	33.7%^b^	37.1%^b^	38.3%^b^	20.9%c	<0.001
	(5.2%)	(5.9%)	(11.8%)	(8.6%)	(0.9%)	(2.9%)	
**Ectoplacental cone/ Junctional Zone**	73.1%^a^	72.0%^a^	13.3%^b^	3.3%^c^	4.3%c	5.1%^a^	<0.001
	(8.5%)	(3.5%)	(4.8%	(2.8%)	(1.1%)	(1.3%)	
**Lower half of Labyrinth**			15.7%^a^	11.3%^a^	15.9%^a^	8.3%^b^	<0.001
			(4.4%)	(3.9%)	(2.0%)	(1.9%)	
**Upper half of Labyrinth**			15.5%^a^	13.7%^a^	17.8%^a^	9.5%^b^	<0.001
			(6.7%)	(3.6%)	(3.9%)	(2.6%)	
**PHH3 as percent of K18-positive cells**	**E8.5**	**E10.5**	**E12.5**	**E14.5**	**E16.5**	**E18.5**	**ANOVA** **(*P*)**
**Chorion/ Chorioallantoic plate**	7.0% ^a^	4.8% ^b^	3.6% ^c^	3.0% ^d^	2.1% ^e^	1.0% ^f^	<0.001
	(1.7%)	(1.4%)	(1.2%)	(1.3%)	(1.0%)	(1.0%)	
**Ectoplacental cone/ Junctional zone**	3.0% ^a^	3.7% ^b^	0.5% ^c^	0.4% ^c^	0.1% ^c^	0.3% ^c^	<0.001
	(1.9%)	(2.1%)	(0.2%)	(0.5%)	(0.2%)	(0.4%)	
**Lower Labyrinth**			3.7% ^a^	1.0% ^b^	0.8% ^b^	0.7%^b^	<0.001
			(0.7%)	(0.6%)	(0.8%)	(0.7%)	

Between E12.5 and E14.5, there was a decrease in both the trophoblast cell number in the chorion, from 1.3 X 10^6^ to 5.6 X 10^5^ (Fig 1B), reflecting a transition of cells from the chorion into the labyrinth compartment, and the percentage of cells in the cell cycle (Table 1). However, the frequency of cell cycle marker expression remained higher in the chorion compared to the other zones in the placenta. In the chorion, Ki67 expression was detected in 21% to 34% of trophoblast cells between E12.5 and E18.5, a rate that was 2- to 5-fold higher than the other zones in the placenta (Table 1). Similarly, the proportion of phospho-histone H3 positive cells was substantially higher in the chorion compared to the other zones in the placenta (Table 1). While the vast majority of the chorion cells in the cell cycle lay along the chorionic plate, there were also isolated islands of chorion-type cells in the labyrinth. Morphologically, these cells were small, cuboidal, and densely packed and thus resembled cells of the chorion proper.

It was apparent that many of the phospho-histone H3-positive cells in the placenta were not K18-positive and therefore were not trophoblast cells. Examination of histological sections at E15.5, a time when the number of phospho-histone H3/K18 double-positive trophoblast cells had declined to almost zero, showed that 45±10% of phospho-histone H3-positive cells were PECAM-positive endothelial cells and 38±6% were α-smooth muscle actin-positive pericytes (S2 Fig). Phospho-histone H3-positive fetal blood cells were also occasionally detected in blood vessels in the placenta.

### Endoreduplication and hypertrophy in trophoblast cell populations in the placenta

**Fig 2:**
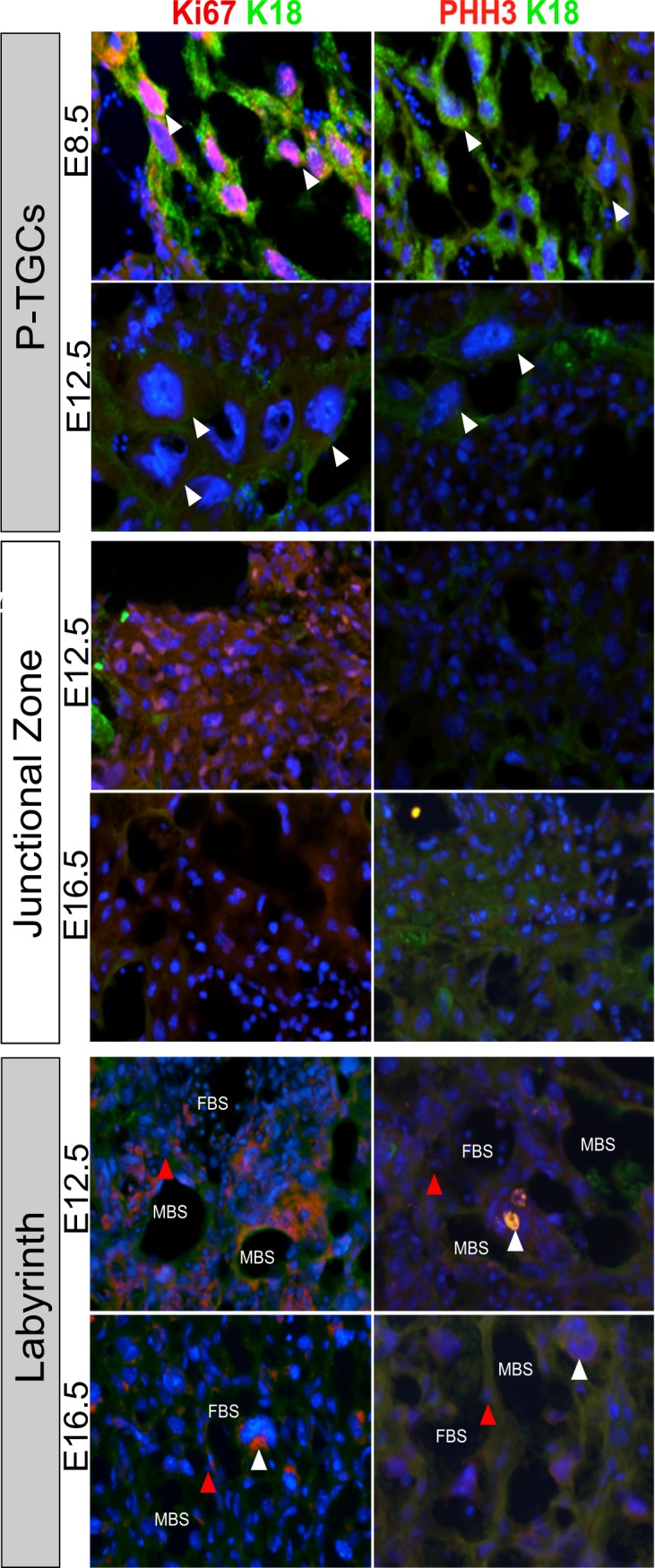
Ki67 and Phospho-histone H3 immunofluorescence staining in P-TGCs, junctional zone and labyrinth. Histological sections were dual stained for Ki67/K18 (red/green respectively) or phospho-histone H3/K18 (red/green respectively) and nuclei are counterstained with DAPI (blue). Phospho-histone H3 was detectable at E12.5 in sinusoidal-TGCs within the labyrinth (white arrow; x400 magnification in the inset) but not in SynT cells (red arrow). MBS–maternal blood space; FBS–fetal blood space.

By comparing the patterns of Ki67 and phospho-histone H3 staining, it was possible to identify populations of trophoblast cells that were undergoing endoreduplication (Ki67-positive but phospho-histone H3-negative) (Fig 2). For example, almost all P-TGCs expressed Ki67 at E8.5 but none expressed phospho-histone H3, indicating that this cell population was not mitotic but, rather, was endoreduplicating (Fig 2). Ki67 was not detected in P-TGCs after E12.5 indicating that they do not continue to endoreduplicate past this time point. While there was some evidence of cell proliferation early, phospho-histone H3-positive cells were nearly undetectable in the junctional zone at E12.5 and E16.5 (Table 1 and Fig 2). The total number of cells that were Ki67-positive also dropped after E12.5 (Table 1 and Fig 2). These data imply that, like TGCs, SpT and/or GlyT cells in the junctional zone endoreduplicate to become polyploid early in pregnancy.

Within the labyrinth layer, the thin SynT cells showed little, if any, phospho-histone H3 or Ki67 staining at any point throughout gestation (Fig 2, red arrows). S-TGCs were distinguished from SynT cells in the labyrinth based on their large cell and nuclear size, K18 staining, and proximity to maternal blood spaces in the labyrinth. S-TGCs showed Ki67 staining at E16.5 (Fig 2, white arrows) in support of previous observations that these cells become polyploid [12,50], presumably as a result of endoreduplication. In contrast to P-TGCs, there were a small number of phospho-histone H3-positive S-TGCs in the labyrinth (Fig 2, white arrow). However, upon close inspection of the staining patterns, we did not observe any evidence of metaphase plates or anaphase spreads in these cells. This indicated that the cells were entering G2 phase of the cell cycle and beginning to condense their chromatin but were not entering M-phase.

Analysis of the labyrinth layer suggested that it undergoes complex growth regulation. Consistent with the decline in trophoblast cell proliferation in the chorion and labyrinth, the total number of trophoblast cells in the labyrinth remained relatively constant after E12.5 (Fig 1C). Despite this, the labyrinth zone expanded in volume by approximately 4-fold linearly between E12.5 to 18.5 (Fig 1C). Previous studies have shown that the total volume of the trophoblast cell compartment in the labyrinth layer similarly increases after E12.5, and plateaus by E16.5 [11]. Based on these data, we calculated the trophoblast cellular volume (normalized to trophoblast nuclear number) and found that it increased by ~5-fold between E12.5 and E18.5 (Fig 1D). These results indicated that the increase in the volume of the trophoblast cell compartment in the labyrinth after E12.5 was driven by trophoblast cell hypertrophy rather than an increase in trophoblast cell number.

### Chronic protein restriction results in sexually dimorphic fetal and placental growth trajectories

**Fig 3:**
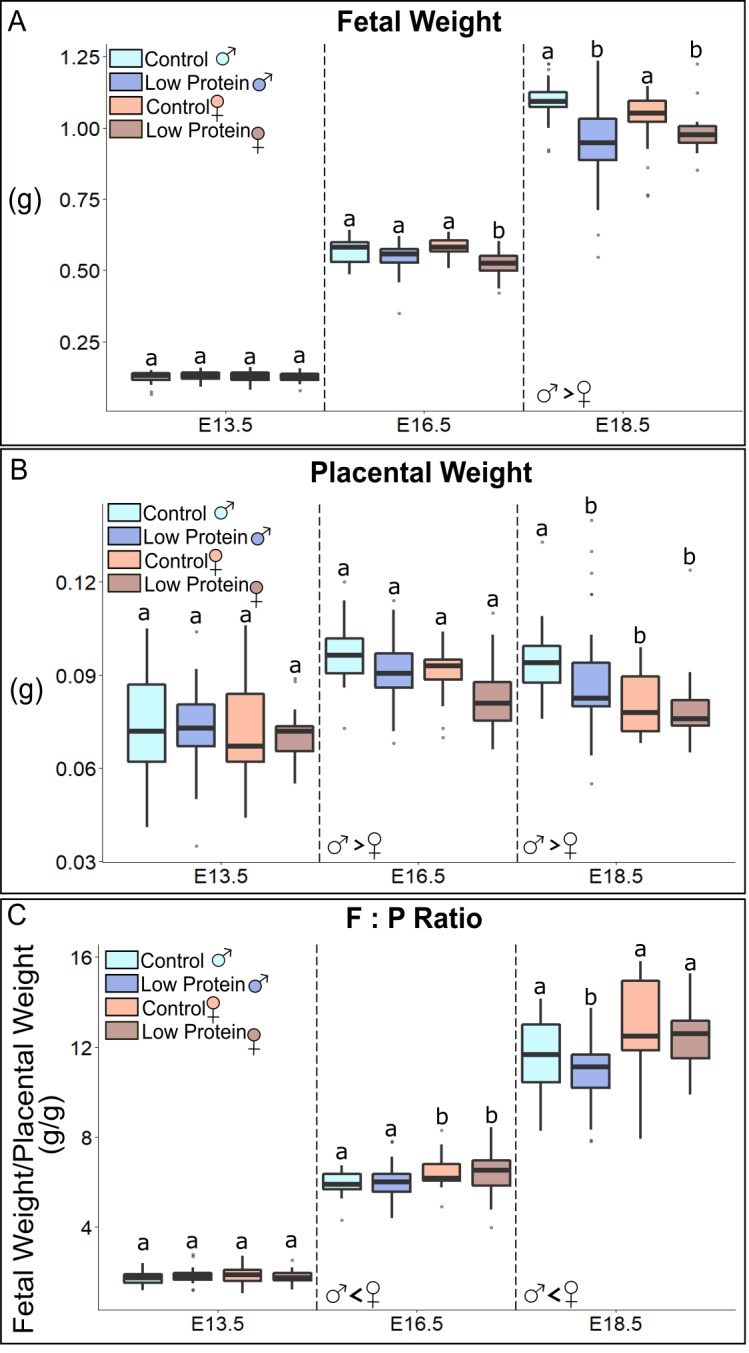
Weights of fetuses and placentas, and fetal/placental weight ratios, in control and dietary protein restricted pregnancies. (A) Fetal weights separated by sex and embryonic day (E). (B) Placental weights separated by sex and embryonic day. (C) Fetal to placental weight ratio (F:P) separated by sex and embryonic day. Explanatory variables–diet, sex, litter; Co-variates–maternal weight, litter size. Letter labels denote statistically significant main effects of diet *within* days (p<0.05). Main effects of sex (if present) are denoted by symbols at respective days (p<0.05). No diet by sex interactions were present. Number of conceptuses in each group (from left to right): E13.5–28, 32, 41, 39; E16.5–24, 37, 26, 24; E18.5–23, 42, 19, 32.

Having established the pattern of cell growth in normal placentas, we next assessed the effects of dietary restriction. To do this, eight-week-old nulliparous female mice were fed either a control (Con) or low protein (LP) diet for two weeks and then paired with normal fed males. We used the C57BL/6 strain based on their use in previous dietary restriction models [28,51]. Based on data described below, C57BL/6 placentas displayed a similar overall growth trajectory as CD1 mice (S3 Fig). Time to mating and litter size did not differ significantly between the control and low protein groups. Pregnant females were sacrificed on embryonic days (E) 13.5, 16.5, and 18.5, the uterus was removed and weights were recorded of individual fetuses, placentas and the non-gravid body of the mother. As expected, non-gravid maternal weight was significantly lower in the LP group compared to the Con group from E13.5 onwards due to maternal weight loss (p<0.001; n = 10/group) (S4 Fig). The degree of maternal weight loss became more severe as pregnancy progressed, with mothers in the LP group being 17%, 23%, and 30% lighter at each respective time point (p<0.001) (S4 Fig). To account for potential variation caused by other factors, regression analysis was performed followed by ANCOVA, with maternal weight and litter size as covariates (S3 Table). Despite severe maternal weight loss from E13.5 onward, fetuses in the LP group were only lighter at E16.5 (p<0.01), and E18.5 (p<0.001) (Fig 3A). Interestingly, female fetuses were more growth-restricted than males at E16.5 (p = 0.03), but not at E18.5.

Because male and female fetuses had different growth trajectories in the LP group, we hypothesized that placentas would have sexually dimorphic growth trajectories as well. There is a significant effect of X chromosome dosage (and hence sex) on placental growth, with male placentas being heavier than female placentas [31]. Consistent with this, the increases in weight and cross-sectional area of the placenta between 13.5 and 16.5 were less in female placentas compared to males, regardless of diet. This resulted in smaller and lighter female placentas at E16.5 (p<0.01) and 18.5 (p = 0.01) (Fig 3B). Placentas in the LP group appeared to be lighter at E16.5 in both sexes. However, the diet effect was only significant in LP males at E18.5 (p = 0.04). Like the cell number and volume in CD1 placentas, C57BL/6 placental weight trajectory was bi-phasic in Con diet group. Looking at the overall trend in the data, we see that dietary protein restriction prevented bi-phasic growth completely in females and flattened the trajectory in males. Because female placentas were already smaller, the diet effect on placental weight was not as severe at E18.5 compared to males. Given this, placental efficiency (driven by size independent factors) was likely shifted, both in the LP diet group and by sex.

Placental efficiency was therefore estimated by dividing fetal weight by placenta weight (F:P ratio) (Fig 3C). Female placentas were more efficient than males, regardless of diet at E16.5 (p<0.001) and E18.5 (p = 0.01). Efficiency in LP males and females was slightly lower than same-sex controls at E18.5 (p = 0.03 and p = 0.04, respectively). At this time point (E18.5) both sexes in the LP group are growth restricted (Fig 3C). Previous work has shown that fetal growth is not dependent on placental weight after E16.5 [52], so we wanted to confirm if there was a significant correlation between fetal and placental weight at E18.5 given the diet effects on placental efficiency. Consistent with the previous study, we found no positive correlation between fetal and placental weight at E18.5 in controls (slope = 0) (S5 Fig). In contrast, there was a positive correlation in the LP groups at E18.5, in males and females (p<0.01), with LP fetal weight increasing by 0.06g per unit increase (0.1g) in placental weight. This indicated a continued reliance of fetal growth on the size of the placenta in the LP group compared to Con (S5 Fig). It remained unknown, however, if fetal sex correlated with fetal weight at E18.5. To answer this question, we separated the effect of sex from that of placenta size, controlling for them separately in the ANCOVA model. Holding fetal weight constant, there was no effect of sex on fetal weight at 18.5, showing that placental weight alone correlates with fetal weight at E18.5. Altogether, the data showed that adaptations in response to low protein diet affecting placental weight had a larger impact at later stages, with sex-specific effects altering growth trajectories and ultimately mediating placental size and efficiency.

### Patterns of growth in the malnourished placenta

**Fig 4:**
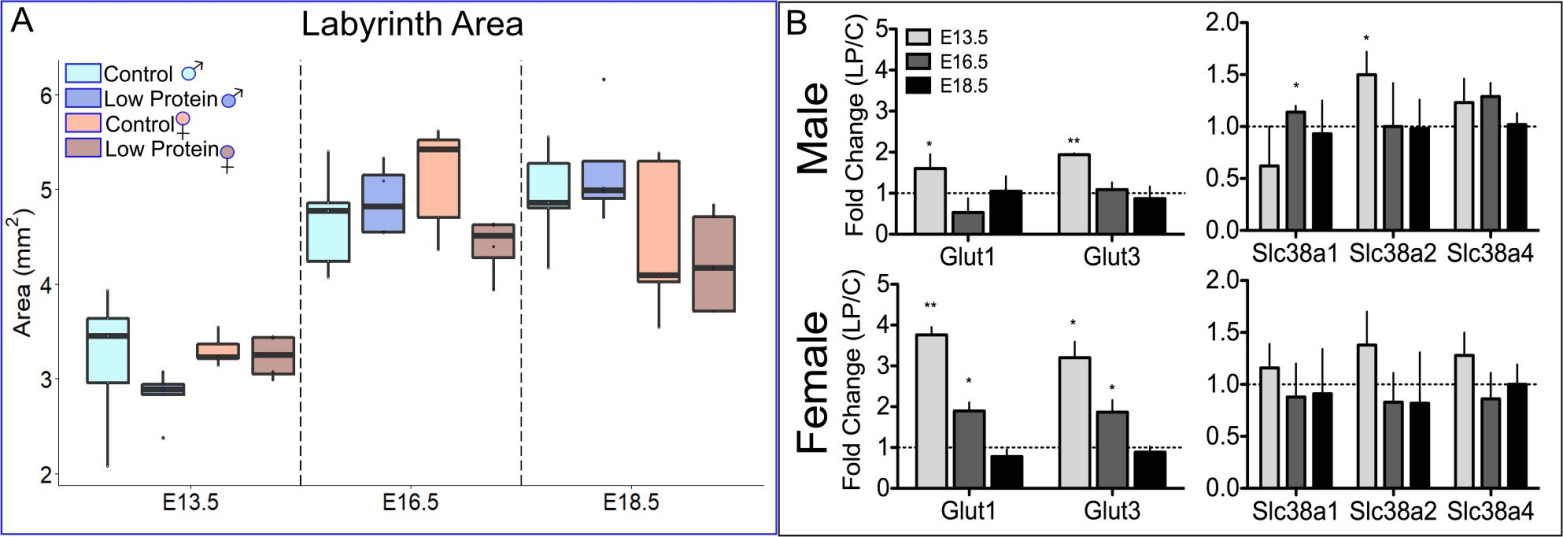
Labyrinth growth in low protein pregnancies is mediated by trophoblast hypertrophy and nutrient transport function. (A) Total labyrinth area separated by diet and sex (n = 4–5). (B) Glucose and amino acid transporter expression in male and female placentas. Fold change in protein restricted placentas (bars) relative to control diet placental expression (dotted line). * p<0.05; ** p < 0.01 (n = 3).

Given its central role in nutrient exchange, we first examined the labyrinth layer, considering both structural (size, vascular composition, cellular composition) and functional (nutrient transporter) characteristics. Previously we found no differences in labyrinth size between control and LP diets [28], but in the current study we found that cross-sectional area was marginally smaller in LP female placentas, compared to LP male placentas at E16.5 (p = 0.05) (Fig 4A). Like placental weight, female labyrinth areas in the LP group did not expand as much as males in the LP group, and overall, female labyrinths were smaller than males at E18.5 (p = 0.02). The composition of the labyrinth was determined through stereological point analysis (Table 2). We used alkaline phosphatase staining to outline the SynT-layer I cells and, therefore, to distinguish cell types including: maternal blood space (MBS), fetal blood space (FBS), S-TGCs, SynT cells, and fetal endothelial cells/pericytes. Largely, diet and sex did not have a significant effect on labyrinth composition between groups at the same stage. One exception was that the relative maternal blood space was lower in female placentas at E13.5 and 16.5, compared to males, in the LP group specifically. Between days, our results showed that the normal expansion of maternal blood space and reduction in fetal blood space were stunted in the LP group, with the maternal blood space in control placentas expanding by ~6%, compared to only ~3% in the LP group.

**Table 2:** Labyrinth composition expressed as a percent area. (mean; SEM in parenthesis).

	E13.5		E16.5		E18.5	
	Control	Low Protein	Control	Low Protein	Control	Low Protein
MBS	17.4%^a^	17.2%^a#^	23.2%^b^	20.9%^a#^	21.4%^a^	24.5%^a^
	(2.1%)	(3.5)	(3.0)	(4.2)	(2.7)	(2.8)
S-TGC	4.0%^a^	5.0%^a^	4.8%^b^	4.5%^a^	4.8%^a^	4.3%^a^
	(0.6%)	(1.5%)	(0.4%)	(1.0%)	(1.2%)	(1.0%)
SynT	18.4%^a^	18.3%^a^	17.9%^a^	22.8%^b^	25.0%^b^	25.4%^b^
	(2.2%)	(2.2%)	(4.7%)	(2.1%)	(1.4%)	(5.0%)
FBS	14.7%^a^	15.1%^a^	11.3%^b^	12.7%^a^	14.5%^a^	14.3%^a^
	(0.6%)	(2.3%)	(1.3%)	(3.2%)	(2.5%)	(2.8%)
Endothelium/	43.6%^a^	41.4%^a^	41.0%^a^	36.9%^a^	32.4%^b^	30.0%^b^
Pericytes	(3.5%)	(3.5%)	(5.0%)	(5.5%)	(5.6%)	(5.4%)

Superscripts indicate statistically different means, p < 0.05.

# indicates a statistically significant (diet: sex) interaction within at embryonic day (lower in females).

In addition to overall size and composition, we looked for functional changes that could explain higher placental efficiency in females. RT-qPCR analysis of several nutrient transporter genes revealed that female placentas expressed higher relative levels of glucose transport genes for a longer duration. Male placentas showed a ~2-fold increase in the levels of glucose transporter genes (*Glut1/3*) at E13.5 in the LP group compared to Con group (p<0.05). Female placentas in the LP group, however, had ~4-fold greater expression of glucose transporter genes compared to Con group at both E13.5 and 16.5 (p<0.05) (Fig 4B). Expression of all three amino acid transporter genes that were assessed (*Slc38a1/2/4*) trended higher in female placentas (p = 0.05) at E13.5, while *Slc38a2* (p = 0.04) and *Slc38a4* (p = 0.06) trended higher in males at E13.5. We also measured the expression of *Igf2 P0* mRNA, the labyrinth specific *Igf2* transcript that has been shown to regulate maturation and functional adaptations in the labyrinth [53–55]. *Igf2 P0* mRNA expression was higher in LP placentas compared to controls at E13.5 and 16.5, in both males and females (S6 Fig).

**Fig 5:**
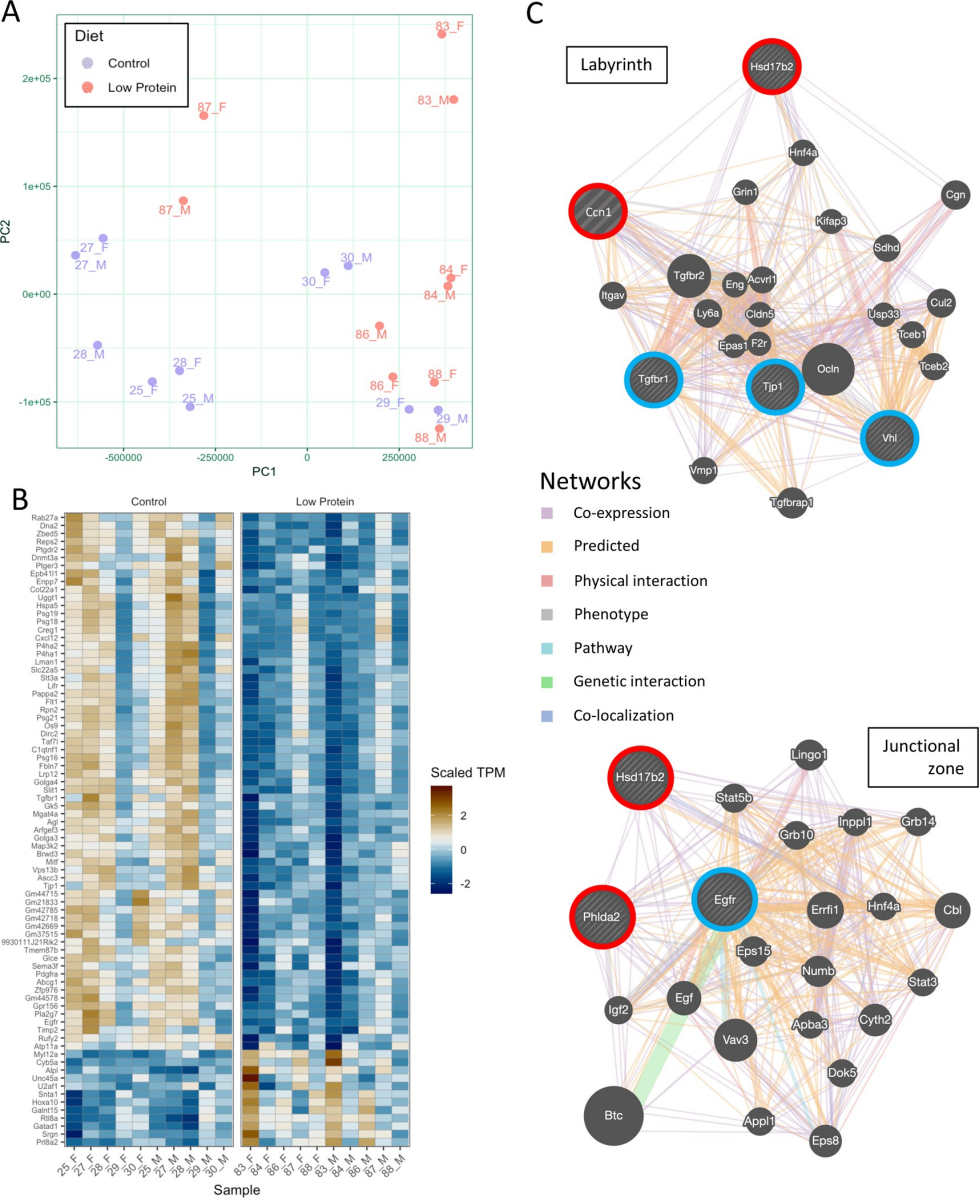
RNA sequencing analysis of Con and LP placentas at E16.5. (A) Principal component analysis with different points representing different litter (number) and sex within litter (male versus female). (B) Heat map of differentially expressed genes. (C) Gene network diagrams produced by GeneMania program, showing relationships between genes that are up- (red) or down-regulated (blue) in LP placentas (n = 5).

To gain further insights into how the development and/or function of the placenta was affected by LP diet, we performed RNA seq analysis using RNA samples from E16.5 placentas (Fig 5, supplementary data). We found that 72 genes were differentially expressed between Con and LP groups (q<0.05). Principal component analysis showed significant segregation of Con and LP placentas but no clear separation of male and female placentas (Fig 5A). There was a significant effect of litter, indicating variation between pregnancies in different females, but what accounted for this was unclear. The most differentially expressed genes came from a variety of different classes (Fig 5B). To determine if specific developmental pathways were affected, we examined expression of genes that have been annotated in the Mouse Genome Informatics (MGI) Phenotype database as having mutant phenotypes with changes in labyrinth and/or junctional zone morphology (Fig 5C). Five genes associated with changes in labyrinth morphology (MP: 0001716) were differentially expressed, with *Ccn1* and *Hsd17b2* mRNA being higher in the LP group, while *Tgfbr1*, *Tjp1* and *Vhl* were expressed at lower levels. The small number of genes associated with these processes is likely a result of the log-fold changes being small across the entire data set, as well as the strong, litter clustering that unfortunately masked sex-specific effects as well. The results of our gene set enrichment test confirmed this.

### Delayed spongiotrophoblast hyperplasia and hypertrophy in malnourished placenta

**Fig 6:**
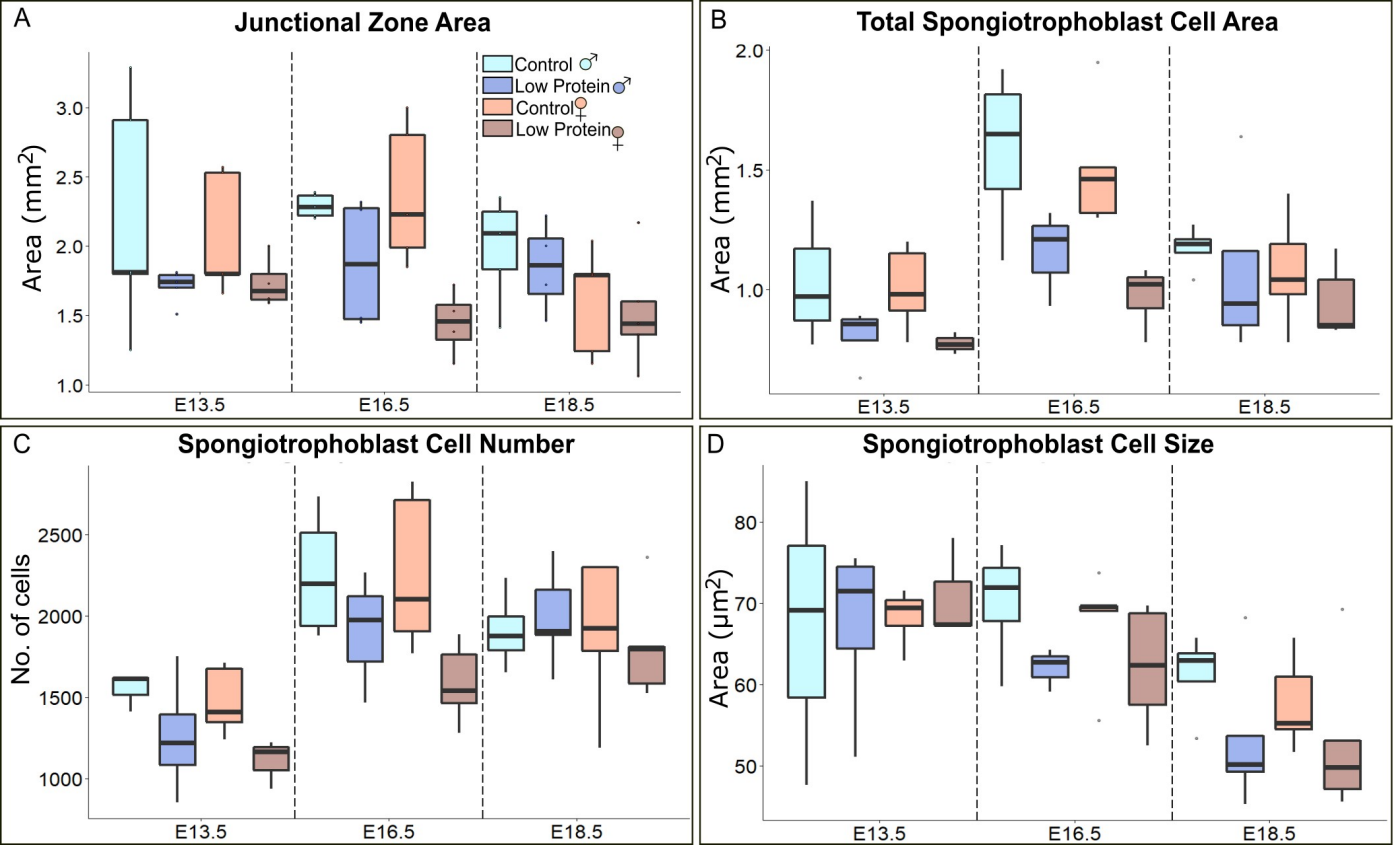
Spongiotrophoblast (SpT) cells contribute to the reduction in junctional zone size due to changes in cell size and number. (A) Junctional zone area. (B) Total SpT cell area was measured in Image J using automated thresholding, distinguishing SpT cells by expression of *Prl8a8* mRNA. (C) Spongiotrophoblast cell number (n = 4–5).

In addition to changes in the labyrinth, maternal dietary protein restriction resulted in a smaller junctional zone area, similar to the results of our previous study [28]. We found that this was true in both sexes at E13.5 (p = 0.04, Kruskal-Wallace) and 16.5 (p<0.01) (Fig 6A). Sex-specific effects were apparent at E16.5 onward. At E16.5, female LP junctional zone area was 42% smaller in females, and 20% smaller in males compared to same-sex controls (Fig 6A). At E18.5, female junctional zones were significantly smaller than males in both diets (p<0.001). The diet effect was less pronounced in females at E18.5 since female junctional zone area drops significantly (p = 0.01) between E16.5 and 18.5 in the Con group. Male junctional zone area was steady across time points and consistently smaller in the LP group.

We next wanted to determine which cell type was contributing to these effects. We quantified total SpT cell area and number, dividing these measures for an estimate of cell size. In the Con diet, total SpT cell area (*Prl8a8* mRNA positive area) was bi-phasic, increasing by 33% from E13.5 to 16.5 and then decreasing by 26% from E16.5 to E18.5 in both sexes (Fig 6B). Increased cell number was a driver of growth between E13.5 and 16.5 in controls. In the LP group, there were consistently fewer cells at E13.5 (p = 0.02) and 16.5 (p = 0.01). The LP group was closer to controls at E18.5 because of a decrease in cell number in Con diet, rather than an increase in the LP group. (Fig 6C). Change in cell size was a clear driver of the reduction in total area, with SpT cells smaller at E18.5 compared to E13.5 in all groups. Given that most of the cell proliferation within the junctional zone occurs before E13.5, our results suggest that protein restriction delays SpT cell differentiation from precursor cells, resulting in fewer *Prl8a8*-positive cells at E13.5. In addition, SpT cells were marginally smaller in the LP group at E16.5 (p = 0.05) and 18.5 (p = 0.09), (Fig 6D) suggesting that delayed differentiation also delays the growth of these cells. In all groups, cell size trended downward from E16.5 to 18.5, (Fig 6D) suggesting either that larger cells were dying, or cells were becoming smaller after E13.5. Cell number and size were similarly affected in males and females.

The other major cell type in the junctional zone is GlyT cells. It is assumed that GlyT cells accumulate glycogen in large vacuoles to buffer energy requirements later in pregnancy. Storage and release of glycogen has previously defined the bi-phasic growth trajectory of the junctional zone [10]. Our measurements of this cell type showed a clear bi-phasic growth trajectory, with the increase in the total area of protocadherin 12 (*Pcdh12*) expressing cells from ~ 2.5 mm^2^ at E13.5 to ~3.5 mm^2^ at E16.5 and decreasing to less than 2.5 mm^2^ at E18.5 in controls (S7 Fig). Interestingly, GlyT cell area in the LP group failed to increase between E13.5 and 16.5, resulting in a downward trend over time and significantly less *Pcdh12* positive area in both sexes at E16.5 compared to the Con group (p = 0.03). Females in both diet groups had significantly less GlyT cell area compared to males at E18.5, but again, the normal reduction in cell area brought controls down to LP levels, so there was no diet effect visible at E18.5. *Pcdh12* mRNA expression was reduced in LP males compared to Con males at E13.5 and was increased in LP females compared to Con females at E13.5 and 16.5, not matching the trend in total cell area (S7 Fig). To assess glycogen cell function, we measured the expression of several genes encoding glycogen synthesis enzymes. Interestingly, LP female placentas expressed significantly less mRNA for gene encoding G*be1* (glycogen branching enzyme) at E13.5 and 16.5 (S6 Fig). Higher levels of glucose transporter expression in females and lower expression of glycogen branching enzyme suggested that more glucose was being transported to the fetus, with less available to accumulate in GlyT cells.

Examination of the RNA seq data showed three genes associated with junctional zone morphology that were differentially expressed (Fig 5C). Of the 106 genes associated with abnormal junctional zone morphology in the MGI Phenotype database (MP:0008957), we found that *Phlda2* and *Hsd17b2* expression levels were increased, while only *Egfr* was reduced in LP placentas (p <0.05; q < 0.1).

### Sinusoidal TGC and spongiotrophoblast cell endoreduplication in protein-restricted pregnancies

**Fig 7:**
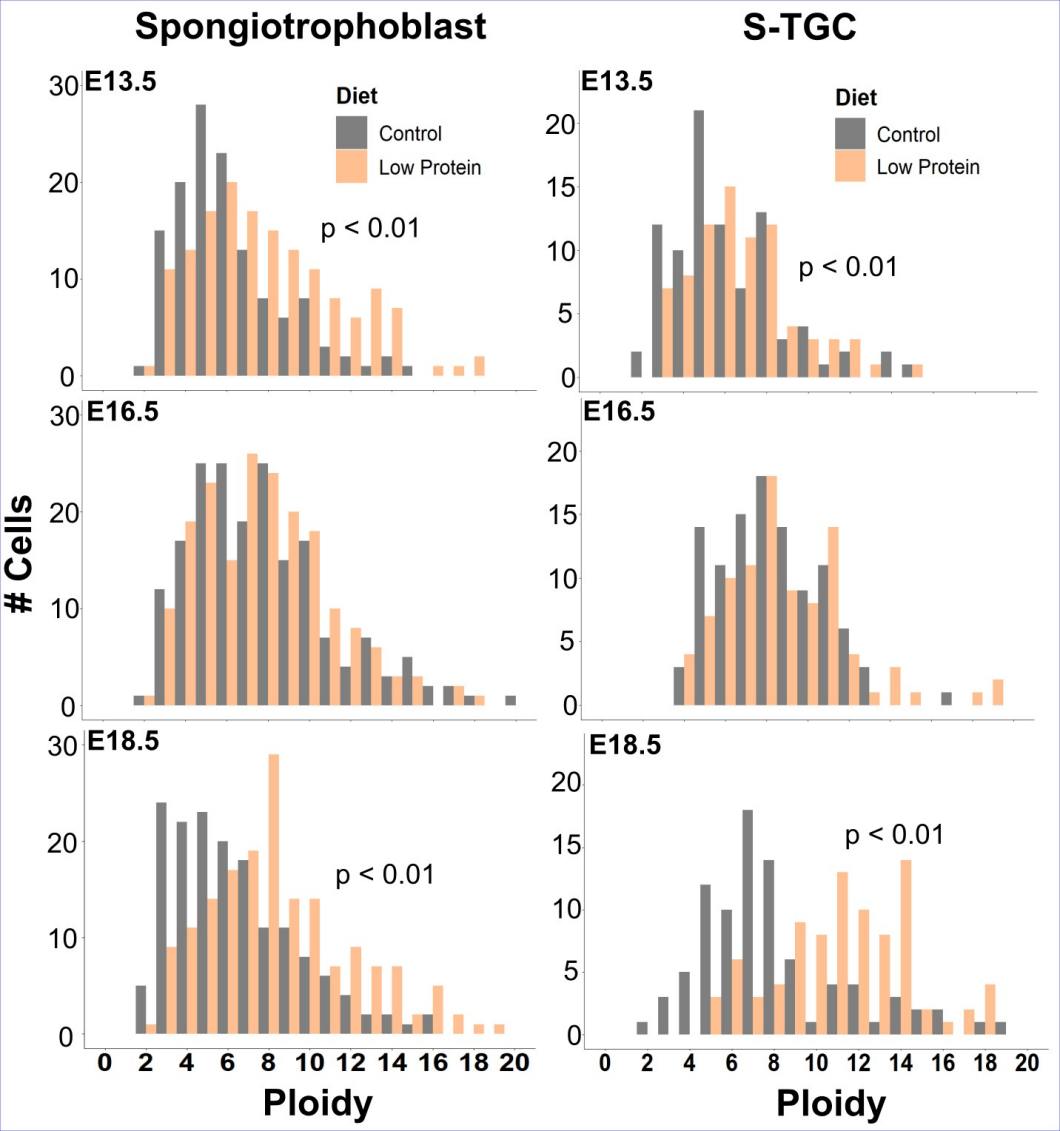
Sinusoidal trophoblast giant cell (S-TGC) and spongiotrophoblast (SpT) cell ploidy in control and protein-restricted pregnancies. (A) Frequency of S-TGCs at each ploidy level across gestation. (B) Frequency of SpT cells at each ploidy level across gestation (n = 6).

We next wanted to see if dietary protein restriction altered the DNA content in polyploid trophoblast cells. We estimated DNA content in S-TGCs, P-TGCs and SpT cells using DAPI fluorescence. As expected from our Ki67 results, P-TGC ploidy remained unchanged from E13.5 to 18.5 (S8 Fig), and diet had no effect. Mean S-TGC ploidy increased in Con and LP placentas from E13.5 (6.12C ± 2.6) to 16.5 (8.20C ±2.9) (Fig 7), consistent with low levels of ongoing Ki67 expression. Interestingly, S-TGC ploidy continued to increase in the LP group, attaining a mean ploidy of 11.24C ± 3.19 at E18.5, compared to Con cells that remained near 8C (mean = 7.96C ±3.4) at E18.5 (p<0.01). If a cell has gone through a third doubling of its entire genome, it would be 16C, but in this case, the data suggests incomplete replication of genomic DNA. SpT cells had a higher ploidy in the LP group compared to Con, evident at both E13.5 and 18.5 (p<0.01). Specifically, cells in the LP group attained a mean ploidy of 9.03C at E18.5 compared to the Con group with a mean ploidy of 6.43C (p<0.01). Some cells in the Con group did attain a ploidy of ~8C at E16.5. However, this population did not persist at E18.5. Sex differences were not significant.

## Discussion

This study quantified how different trophoblast cell types contribute to the growth of the placenta during normal development and under conditions of severe dietary restriction. First, we used stereology to quantify cell numbers, focusing on development after E8.5 when hyperplasia drives rapid expansion. Placental growth after E12.5 is then driven by trophoblast cell hypertrophy and endoreduplication in several other trophoblast cell types. We then fed mice a chronic low protein diet and observed if and how placental structure and/or function compensated. Despite severe maternal weight loss in the protein restricted group, fetal growth was largely preserved until late gestation and interestingly, males and females displayed different growth trajectories. While female placentas were able to make up for their small size by becoming more efficient, male placentas compensated through labyrinth zone expansion. We then showed that SpT cell differentiation and growth is altered by protein restriction, contributing to the late-term loss of mass in the junctional zone. Finally, we showed that protein restriction causes S-TGCs and SpT cells to attain a greater ploidy at E18.5 compared to controls.

### Patterns of growth in the normal placenta

The developmental events at E8.5 mark a major transition in the placenta. The allantois contacts the chorion, and together they give rise to the labyrinth layer. At the same time, the ectoplacental cone expands to give rise to the junctional zone [12]. Our current study showed that these events were accompanied by large increases in the number of trophoblast cells/nuclei in both the chorion and ectoplacental cone between E8.5 to E12.5. In order to identify specific cell populations that proliferate, we used markers that allowed us to distinguish true cell proliferation from endoreduplication, the process in which cells replicate DNA but do not go through mitosis and cell division. Our data showed that while trophoblast cells in both the chorion and the ectoplacental cone replicate, the rate is higher in the chorion, which presumably supports the more extensive growth of the labyrinth compared to the junctional zone. After E12.5, while there were a small number of Ki67-positive as well as phospho-histone H3-positive cells in the labyrinth, the majority of these cells were either pericytes or fetal endothelial cells. After E12.5, we found that despite the predicted influx of labyrinth precursors from the chorion, the number of trophoblast nuclei in the labyrinth remained reasonably constant. This apparent paradox could be explained by trophoblast cell death and turnover in the labyrinth as has been observed in the villi of the human placenta [56], the analogous placenta structure.

After E12.5, we found that the volume of the labyrinth continued to increase towards term, but the growth was due to cell hypertrophy rather than hyperplasia. Because trophoblast cells in the labyrinth fuse to give rise to multi-nucleated SynT cells, we assessed cytoplasmic volume on the basis of the number of trophoblast cell nuclei. While trophoblast nuclear number did not change dramatically between E12.5 and E18.5, there was a linear increase in trophoblast cell volume per trophoblast cell nucleus. Likewise, the absolute volume of the labyrinth continued to increase linearly until E18.5, albeit at a faster rate compared to trophoblast cell volume after E14.5. This indicated that between E12.5 and E18.5, the growth of the labyrinth is driven not only by an increase in cell volume but also by an increase in extracellular space. Whether the increase in extracellular volume is due to loss of cell adhesion molecules [57] and the subsequent separation of cells, an increase in the number and/or diameter of maternal and fetal blood spaces[11,58], or a combination of both processes, remains to be elucidated. Our results demonstrate that maternal blood space does increase significantly between E13.5 and E16.5.

Interestingly, we detected a number of trophoblast cell populations that were Ki67-positive but negative for the phospho-histone H3 marker suggesting that these cell populations were endoreduplicating. There are at least five types of TGCs in the mouse placenta that differ in location, developmental origin and function but share the property of being polyploid [12]. Our current results revealed some differences in the timing and progression of endoreduplication in different trophoblast cell subtypes. For example, P-TGCs endoreduplicate their DNA only up to E12.5. These cells are mononuclear and chromosomes are polytene [59]. Consistent with this, we failed to detect phospho-histone H3 expression in P-TGCs. S-TGCs only become detectable in the placenta after ~E12.5 [12] and, in our current studies, they showed Ki67 expression up to E16.5. The other surprise was that we detected, albeit at a low rate, phospho-histone H3 expression in S-TGCs despite prior evidence that S-TGCs are mononuclear and polyploid [12]. During the mitotic cell cycle, the phospho-histone H3 epitope is expressed in the late G2 phase from the time that chromosomes begin to condense in prophase, through metaphase and anaphase. Close examination of the S-TGCs that were phospho-histone H3-positive showed no evidence of S-TGCs in metaphase or anaphase, consistent with a block to mitosis.

In addition to the various TGCs, our data also suggested that endoreduplication occurs in the junctional zone, consistent with prior observations of polyploidy in the junctional zone in rats and rabbits [59,60]. The junctional zone, which arises from the ectoplacental cone, is composed of both GlyT cells and SpT cells. Intriguingly, Ki67 was detected predominantly in GlyT cells in the junctional zone at E12.5 and these cells did not express phospho-histone H3. This suggested that GlyT cells were not proliferating and instead endoreduplicate their DNA and do so before migrating out of the junctional zone into the decidua (10, 58). The low frequency of Ki67 staining in SpT cells suggested that they reach their maximum ploidy before E12.5 and, subsequently, exit the cell cycle. Previous studies have reported that there is an 80-fold increase in the number of GlyT cells between E12.5 and E16.5, before declining by 60% by E18.5 [10]. However, if these cells are not mitotically active, the increase must be due to the differentiation of precursor cells.

### Sexually dimorphic effects of poor maternal diet on fetal and placental development

Placental development is subject to multiple inputs that stretch its adaptive capacity. Having previously shown that protein restriction in the maternal diet impacts placental growth and maternal weight before fetal growth [28], we furthered this work by separating groups by sex in order to account for diet-induced variability in gene expression between sexes [30,61]. Like our previous study, fetal growth in the low protein group was preserved well after one would expect, given early and severe maternal weight loss that preceded a general reduction in placental weight and junctional zone area. Transient growth restriction in females at E16.5 is a novel finding on the timing of IUGR and the capacity for recovery, showing a quantitative example of males and females employing different growth strategies in utero. As expected, male placentas were larger specifically due to a larger labyrinth area but, despite this, these placentas were less efficient than females after E13.5. A previous study found that lighter placentas have more functional adaptations [32]. However, that study did not link the size difference to sex. Our findings here, that placental weight and not fetal sex impacted fetal growth at E18.5, clarifies this relationship. Given that placental size is determined by X chromosome dosage, we hypothesize that females develop functional adaptations earlier, making them more resilient in the face of size reduction in late pregnancy. Males on the other hand, could be more susceptible to placenta size-dependent factors late in pregnancy but are not initially growth restricted because of a larger surface area. Human cohort studies have shown that males compensate for IUGR with placental growth in late pregnancy [62], a feature present in several of the parameters measured in this study. This type of analysis allows us to pinpoint the source and timing of variation and will inform how placental adaptability and fetal growth trajectories are interpreted going forward. It also raises important questions about what type of placental adaptation would lead to specific post-natal growth variation and susceptibility to disease.

### Low protein diet alters the structure and function of the labyrinth zone

The labyrinth zone was a logical starting point to quantify functional and structural changes in the placenta that adapted to the maternal environment. The labyrinth contains trophoblast-lined maternal blood sinuses and fetal vasculature that are highly responsive to external factors such as oxygen availability and Igf2 [53,63]. *Igf2 P0*, a well-established regulator of labyrinth maturity and growth [53], was up-regulated in the LP groups. Giving that male labyrinths increased their size, where females did not, suggests that there could be a sex-specific response to *Igf2 P0*, providing an increase in growth in males, and an increase in nutrient transporter expression in females. We then found that maternal dietary protein restriction resulted in smaller labyrinth areas in females, specifically at E16.5. Because the initial establishment of the labyrinth zone occurs over a short window, it is an energy intensive process, and it is surprising that the labyrinth can grow normally in male placentas despite severe maternal weight loss. Transient growth restriction in female fetuses is mirrored by transient growth restriction in the labyrinth, but other adaptations like glucose transport are able to rescue sex differences in fetal growth by E18.5. This data suggests then, that male placentas rely on placenta surface area to remain efficient, with the caveat that this type of resiliency alone is not enough to preserve fetal growth to term.

Despite the diet effect on labyrinth size being restricted to E16.5, we also found that the LP diet stunted maternal blood space expansion in the labyrinth. This directly affects the surface area available for nutrient exchange, suggesting that without functional adaptations in place, IUGR would follow this disruption. Our data, therefore, suggest that sex-specific adaptations determine fetal and placental growth trajectories that are established by early differences in placental structure and function.

### Both spongiotrophoblast and glycogen trophoblast cells are affected by low protein diet

An initial goal of this study was to determine why the junctional zone is smaller in protein restricted pregnancies. Normally, junctional zone growth is bi-phasic, increasing from E12.5 to 16.5 and decreasing to term [11]. Cell size and number for both GlyT and SpT cells contributes to this growth trajectory [10], and we wanted to determine if one cell type was preferentially affected by protein restriction. We quantified total SpT cell area and saw that it was reduced at E13.5 and 16.5, reflecting a delay in cell proliferation (cell number) and growth (cell size E16.5). Previous reports show that subcellular complexity of SpT cells increases across gestation, likely resulting in increased transcriptional capacity [50]. Given this, the reduced cell size in the LP group may suggest that SpT cells are less functional. In both Con and LP diets, SpT cells don’t increase in size after E13.5 but their number does increase, contributing to the growth of the junctional zone. This appears to be a difference with CD1 strain mice since junctional zone number did not increase (Fig 1). Across both sexes, SpT number and size was reduced by the low protein maternal diet. The other major cell type in the junctional zone, GlyT cells, are only detectable after E12.5 and originate in the ectoplacental cone [64]. After differentiating, many GlyT cells proliferate (E12.5–14.5) and migrate into the maternal decidua [10]. In the LP group, total GlyT cell area failed to increase between 13.5 and 16.5 but still underwent apoptosis after E16.5 [65]. *Phlda2* mRNA expression, which was increased in the LP group at E16.5, is known to restrict the growth of SpT and GlyT cells and reduces the energy storage capacity of the placenta [14]. Epidermal growth factor receptor (*Egfr*) gene expression was reduced in LP placentas as well. *Egfr* knockout mice have restricted junctional zone growth, but this does not account for embryonic lethality in these mutants [66].

### Trophoblast cells endoreduplicate, responding to protein restriction by increasing DNA content

Polyploidization is a well-known feature of TGCs in the placenta but its physiological function is unknown [67]. After shedding light on the mechanisms responsible for the transition into the polyploid state [68], it was discovered that P-TGCs have loosely packed chromatin [69], and selectively over-amplify specific regions of their genome [20]. Interestingly, these regions contain genes that encode placenta prolactin-related hormones. This evidence supports the theory that polyploidization is a low-cost way to increase gene copy number without intervening mitosis and cytokinesis, which makes sense for the placenta that develops quickly [18]. In this study, we used cell cycle markers to show that several trophoblast cell types, not just P-TGCs, exit the mitotic cycle but endoreduplicate. S-TGCs line maternal blood spaces in the labyrinth [15] and are directly exposed to varying levels of oxygen and nutrients, depending on their exact position in the labyrinth [58,70]. Interestingly, S-TGCs in the LP group had a higher DNA content than controls at E18.5. The trend suggests that S-TGCs in a normal fed placenta become smaller or die between E16.5 and 18.5, whereas cells in the LP group continue to increase their DNA content. Our group previously showed that *Prl3d1* mRNA expression in S-TGCs was not affected by short-term fasting [71]. However, ablation of these cells resulted in single-transgenic litter mates dying, suggesting that the endocrine function of these cells is critical for fetal development [72].

SpT cells produce the widest variety of placental prolactin-related hormones. In control conditions, these cells cease endoreduplication after E12.5. However, SpT cells in the low protein group had a higher DNA content compared to controls at E13.5 and 18.5 indicating that they continue to endoreduplicate. Our study only measured total DNA content, but we know that selective regions are over-and under-amplified in P-TGCs at least [20]. Given that *Prl3a1* mRNA expression is elevated in protein-restricted pregnancies up to E17.5 [28], endoreduplication may be the mechanistic determinant of an increase in hormone expression.

## Conclusion

Together, our data reveal the complexity of cellular development in the placenta during normal and perturbed pregnancies. This work will support further studies on the differentiation, proliferation, and adaptive responses seen in trophoblast cells and how they affect fetal growth. The programming power of the placenta necessitates detailed time-course analysis of growth trajectories to distinguish between pathological and adaptive responses. Doing this will lead to a deeper appreciation for the dynamic nature of placental development to better predict and treat pregnancy complications that support fetal development.

## Supporting information

S1 Fig Image of alkaline phosphatase/nuclear fast red-stained labyrinth zone (40x magnification) with superimposed dot-grid and arrows indicating cell types and vascular structures. Two blinded observers quantified 100 dots/image on n = 4 labyrinth images from n = 4 placentas in each group. The cell type or vascular structure underlying each dot was quantified. S-TGC = Sinusoidal Trophoblast Giant Cell; SynT = Syncytiotrophoblast; MBS = Maternal Blood Space; FBS = Fetal Blood Space.
(TIF)

S2 Fig Phospho-histone H3 immunofluorescence staining in pericytes and endothelial cells. Phospho-histone H3-positive pericytes and endothelial cells are present in the labyrinth zone of the placenta at E15.5. Double immunofluorescent staining of αSMA/phospho-histone H3 and PECAM/phospho-histone H3 marks dividing pericytes and endothelial cells respectively. Nuclei are counterstained with DAPI (blue). Scale bar = 100 μm.
(TIF)

S3 Fig Total placental area in C57Bl/6 mice, separated by diet, sex, and embryonic day (E). Three non-adjacent sections from n = 3 biological replicates in each group were manually traced in image J. Symbols represent the direction of the main sex effect (if present). A main effect of diet was only observed at E16.5.
(TIF)

S4 Fig Maternal weight trajectories in control and protein restricted pregnancies (n = 10). (TIF)

S5 Fig Fetal weight as a function of placental weight at E13.5, 16.5 and 18.5 separated by diet and sex. The amount of fetal weight variation explained by placental weight is expressed as adjusted R^2^ values. At E18.5, 0.06g denotes the average fetal growth increase in the LP group per unit change (0.1g) of placental weight (red and blue regression lines). Significant diet:placenta interaction at E18.5 only (p<0.001).
(TIF)

S6 Fig Relative gene expression of zone specific, glycogen synthesis and insulin-like growth factor genes. Expression of junctional (*Tpbpa*), and labyrinth (*Ctsq*) specific genes. *Pcdh12* and *Prl8a8a* are expressed in glycogen trophoblast cells and spongiotrophoblast cells specifically. Four genes involved in glycogen synthesis were measured, glycogen branching enzyme was the only gene significantly different between diets. Insulin-like growth factor-2 and the labyrinth-specific transcript Igf2P0 expressed across diets in males and females. Bars represent log(1+x) fold-change expression in protein restricted pregnancies relative to controls set at 1. *p<0.05; **p<0.01 (n = 3).
(TIFF)

S7 Fig Glycogen trophoblast cell total area in the junctional zone and labyrinth (n = 4–5). (TIF)

S8 Fig Ploidy of P-TGCs at E13.5, 16.5 and 18.5 (n = 4–5). (TIF)

S1 Table PCR primer sequences. (XLSX)

S2 Table Estimates of cell cycle length (in hours) in the chorion and ectoplacental cone/spongiotrophoblast during various stages of gestation. (XLSX)

S3 Table Fetal and maternal weight ANCOVA over three embryonic days. (XLSX)

S1 File *Prl8a8* threshold boost. ImageJ macro used for quantification of DAPI stained nuclei in the junctional zone.
(IJM)
