# Supplementary figures and images for: Complex patterns of cell growth in the placenta in normal pregnancy and as adaptations to maternal diet restriction
Source: PLoS One. 2020 Jan 9;15(1):e0226735. doi: 10.1371/journal.pone.0226735 (PMC6952106; doi:10.1371/journal.pone.0226735)

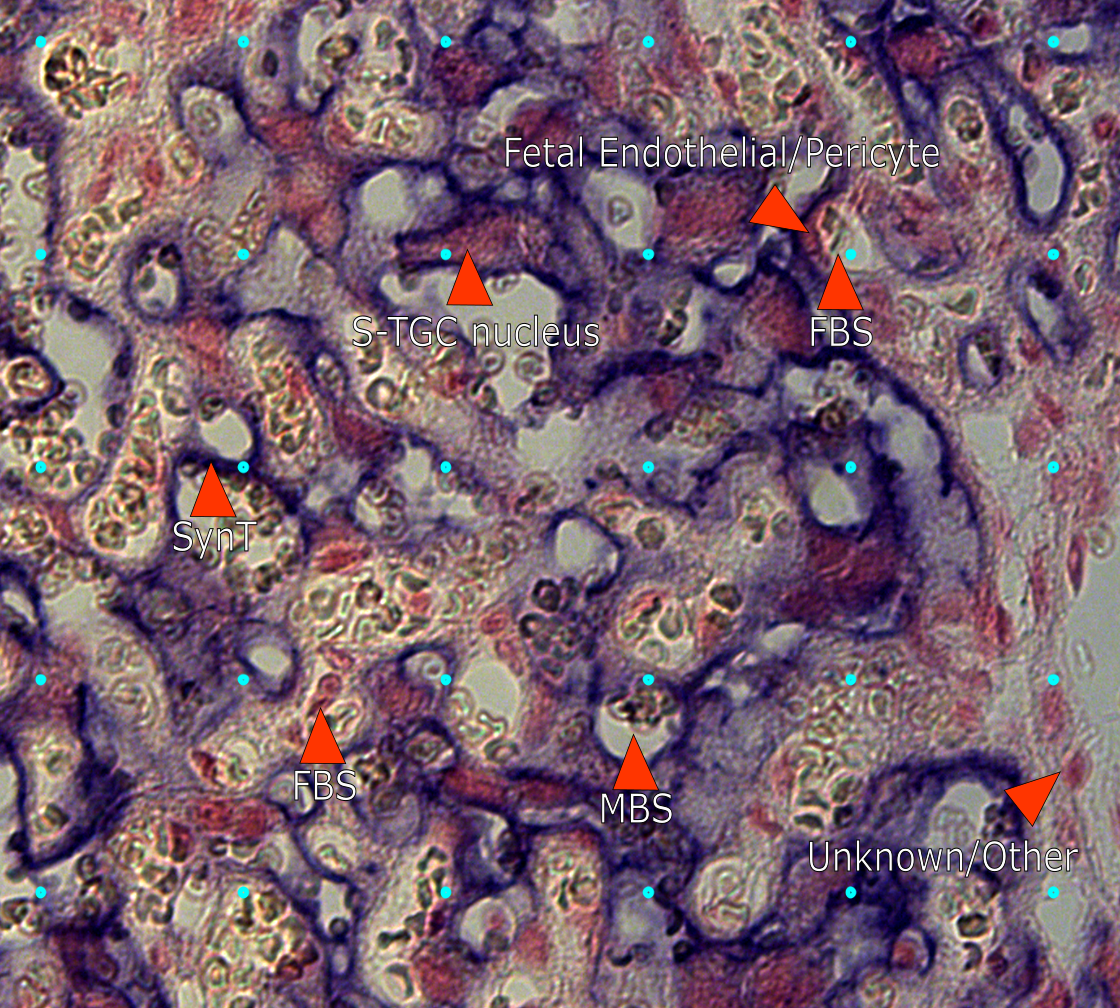

Supplement: S1 Fig — Two blinded observers quantified 100 dots/image on n = 4 labyrinth images from n = 4 placentas in each group. The cell type or vascular structure underlying each dot was quantified. S-TGC = Sinusoidal Trophoblast Giant Cell; SynT = Syncytiotrophoblast; MBS = Maternal Blood Space; FBS = Fetal Blood Space. (TIF) [file pone.0226735.s001.tif]

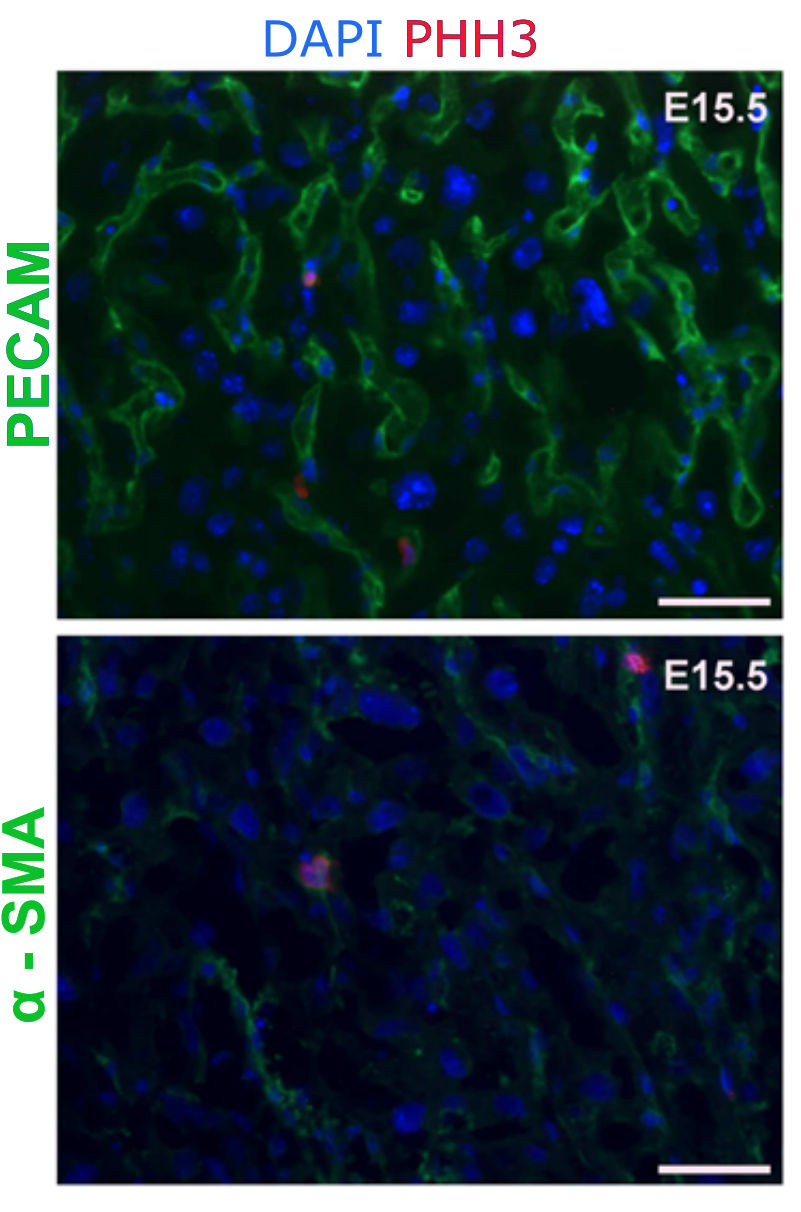

Supplement: S2 Fig — Phospho-histone H3-positive pericytes and endothelial cells are present in the labyrinth zone of the placenta at E15.5. Double immunofluorescent staining of αSMA/phospho-histone H3 and PECAM/phospho-histone H3 marks dividing pericytes and endothelial cells respectively. Nuclei are counterstained with DAPI (blue). Scale bar = 100 μm. (TIF) [file pone.0226735.s002.tif]

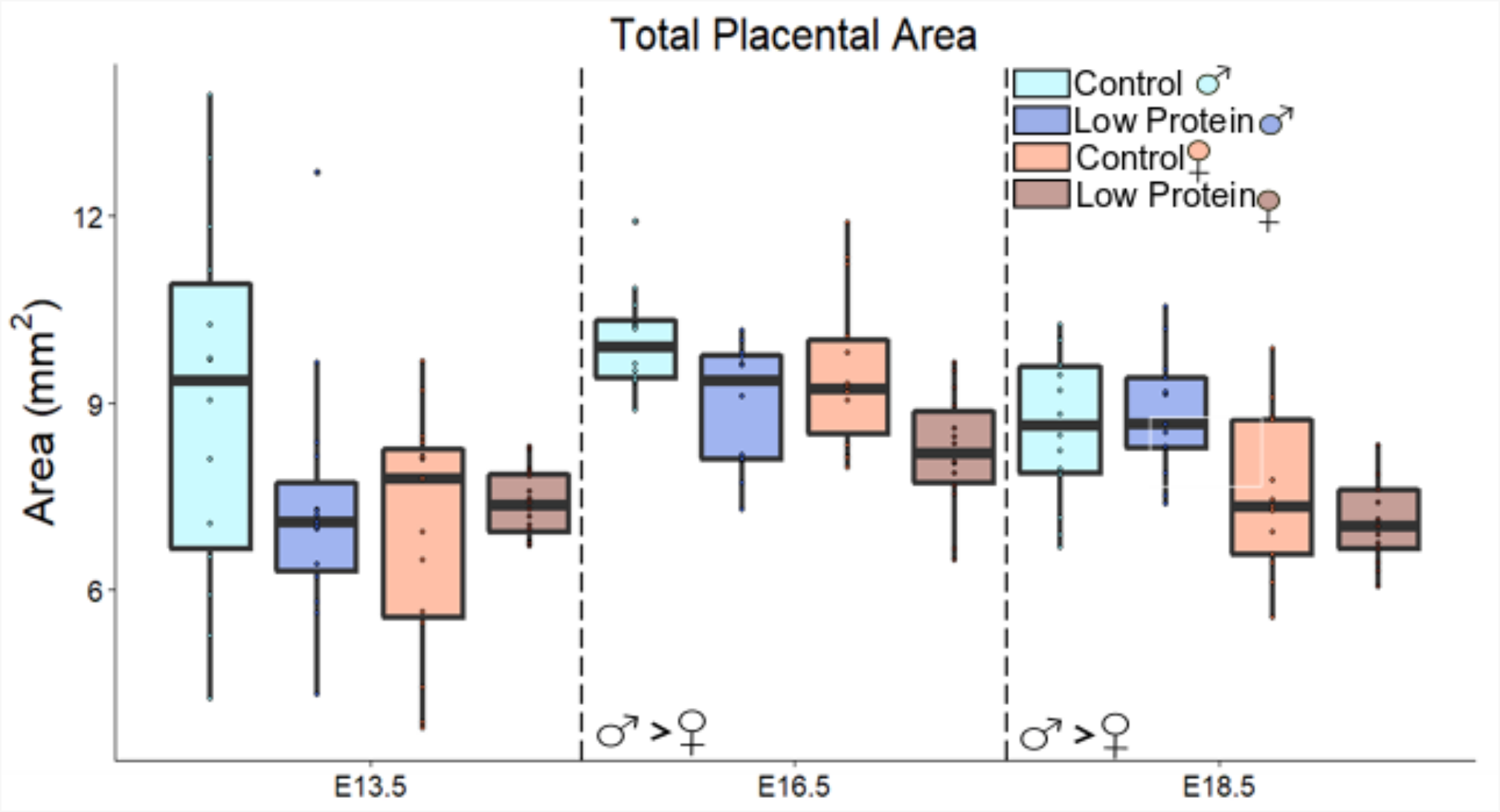

Supplement: S3 Fig — Three non-adjacent sections from n = 3 biological replicates in each group were manually traced in image J. Symbols represent the direction of the main sex effect (if present). A main effect of diet was only observed at E16.5. (TIF) [file pone.0226735.s003.tif]

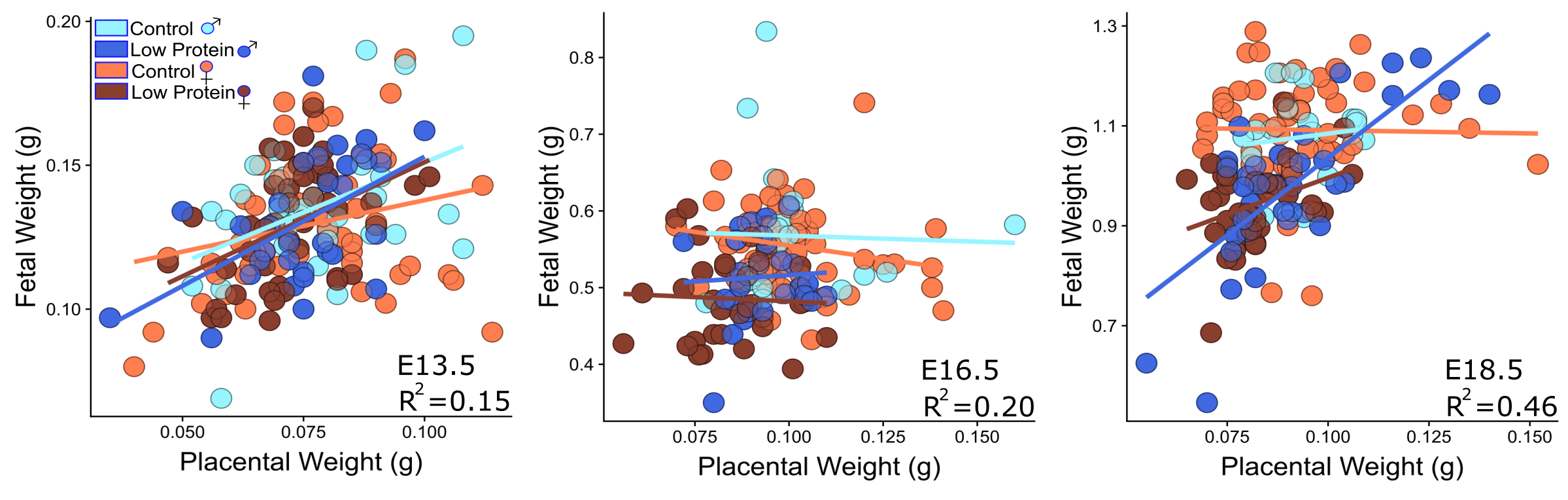

Supplement: S5 Fig — The amount of fetal weight variation explained by placental weight is expressed as adjusted R2 values. At E18.5, 0.06g denotes the average fetal growth increase in the LP group per unit change (0.1g) of placental weight (red and blue regression lines). Significant diet:placenta interaction at E18.5 only (p<0.001). (TIF) [file pone.0226735.s005.tif]

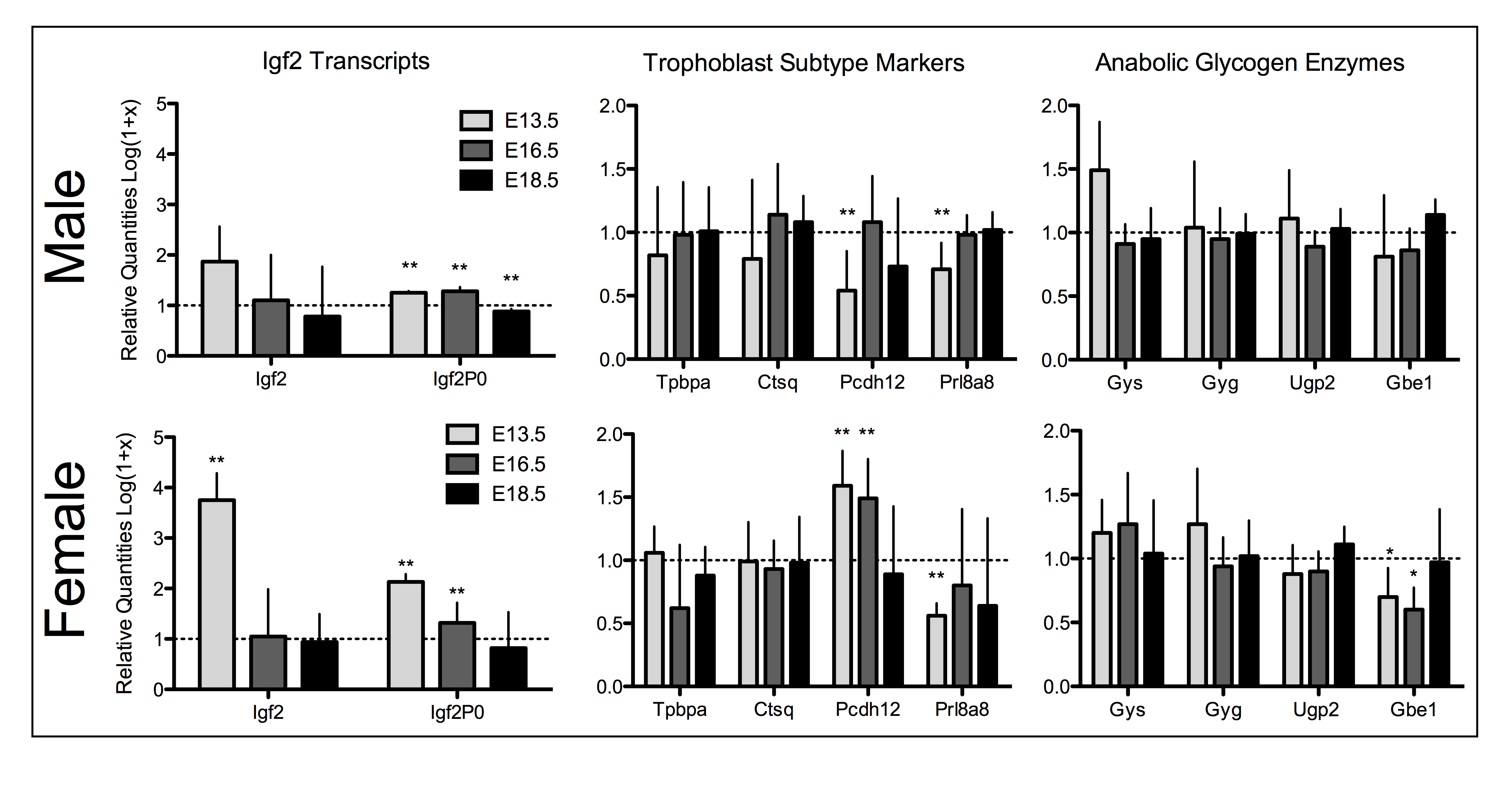

Supplement: S6 Fig — Expression of junctional (Tpbpa), and labyrinth (Ctsq) specific genes. Pcdh12 and Prl8a8a are expressed in glycogen trophoblast cells and spongiotrophoblast cells specifically. Four genes involved in glycogen synthesis were measured, glycogen branching enzyme was the only gene significantly different between diets. Insulin-like growth factor-2 and the labyrinth-specific transcript Igf2P0 expressed across diets in males and females. Bars represent log(1+x) fold-change expression in protein restricted pregnancies relative to controls set at 1. *p<0.05; **p<0.01 (n = 3). (TIFF) [file pone.0226735.s006.tiff]

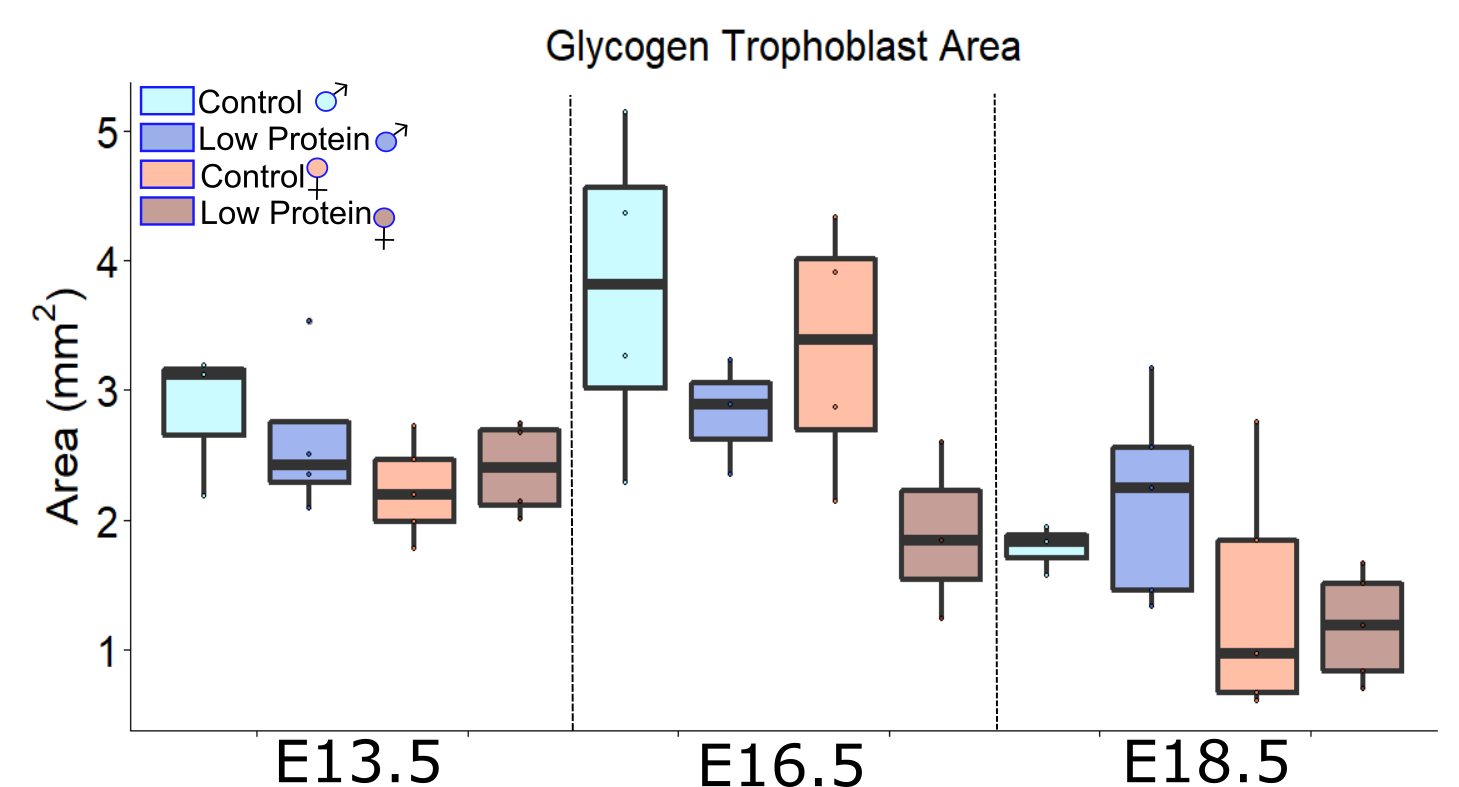

Supplement: S7 Fig — (TIF) [file pone.0226735.s007.tif]
